# Organic Thermoelectric Materials for Wearable Electronic Devices

**DOI:** 10.3390/s24144600

**Published:** 2024-07-16

**Authors:** Runfeng Xiao, Xiaoyan Zhou, Chan Zhang, Xi Liu, Shaobo Han, Canyan Che

**Affiliations:** 1 College of Textile Science and Engineering, Wuyi University, Jiangmen 529020, China; runfengxiao2024@163.com (R.X.); zchan1111@163.com (C.Z.); liuxi@wyu.edu.cn (X.L.); 2 Taizhou Research Institute, Southern University of Science and Technology, Taizhou 317700, China; zhouxiaoyan16@163.com; 3 State Key Laboratory of Luminescent Materials and Devices, Institute of Polymer Optoelectronic Materials and Devices, South China University of Technology, Guangzhou 510641, China

**Keywords:** wearable electronic devices, organic thermoelectrics, generator, sensor

## Abstract

Wearable electronic devices have emerged as a pivotal technology in healthcare and artificial intelligence robots. Among the materials that are employed in wearable electronic devices, organic thermoelectric materials possess great application potential due to their advantages such as flexibility, easy processing ability, no working noise, being self-powered, applicable in a wide range of scenarios, etc. However, compared with classic conductive materials and inorganic thermoelectric materials, the research on organic thermoelectric materials is still insufficient. In order to improve our understanding of the potential of organic thermoelectric materials in wearable electronic devices, this paper reviews the types of organic thermoelectric materials and composites, their assembly strategies, and their potential applications in wearable electronic devices. This review aims to guide new researchers and offer strategic insights into wearable electronic device development.

## 1. Introduction

**Figure 1:**
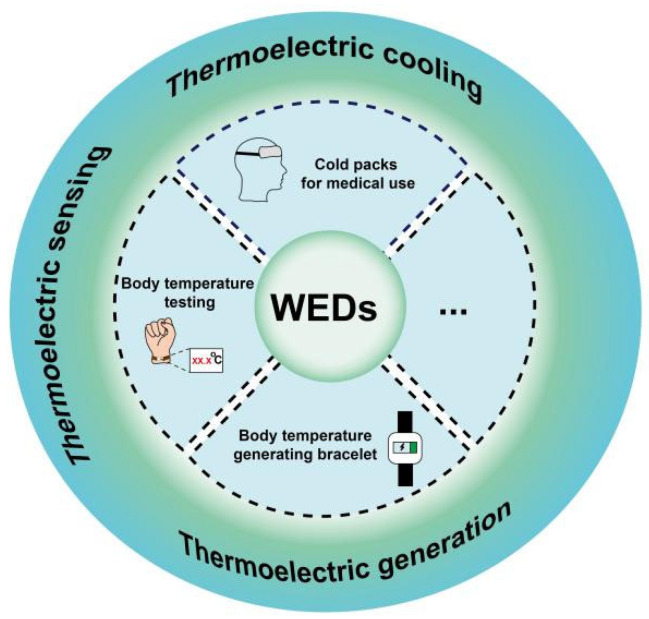
Applications of thermoelectric materials in the field of WEDs.

Wearable electronic devices (WEDs) are a class of electronic devices that come into direct contact with skin or clothing, and are often used to collect human vital signs or expand the capabilities of people with physical disabilities. Therefore, WEDs are considered as one of the research hotspots in the 21st century. In the field of healthcare, WEDs collect body signals such as temperature, heart rate, and blood oxygen saturation, which can help ordinary people in assessing their daily health or assist doctors in making clinical diagnoses. WEDs can also be applied to artificial intelligence-based robots, and to establish human-like perceptual abilities. As shown in Figure 1, here are different kinds of WEDs, including electronic skin [1,2,3], cochlear implants [4], cardiac pacemakers [5], smart glasses [6,7,8], mouth guards [9], and other devices that can monitor, prevent, and even repair damage to human tissue. For instance, Yokota et al. reported a type of organic electronic skin consisting of a three-color, high-efficiency polymer light-emitting diode (PLED) and an organic photodetector (OPD), which was thinner than human skin. This electronic skin is flexible and stretchable, and can visually display the oxygen concentration in blood in real time [1]. This study is of significant importance for the health monitoring of athletes in extreme sports. Han et al. reported a novel implantable pacemaker, which consisted of three parts, namely, an energy harvesting unit, power management unit (PMU), and pacemaker unit [5]. It can effectively correct or prevent cardiac diseases such as sinus arrhythmia. Wang et al. developed a compact wearable device that enables self-sufficient, pseudo-continuous, and low-intrusion tracking of blood glucose levels based on a shape memory alloy (SMA) actuator [10]. Xiao et al. prepared a sensor based on Uircase@MAF-7 using a metal–organic framework (MOF), which can accurately and sensitively detect uric acid (UA) concentrations in sweat. This was achieved by combining a pliable microfluidic chip with a wireless electronic detection unit [11]. As shown by the studies mentioned above, WEDs hold great potential for application in human healthcare.

Besides being employed as sensors to monitor light, temperature, pressure, blood oxygen levels, etc., WEDs are also widely used in energy harvesting systems that convert various types of environmental energy into electricity, based on effects like photoelectric, thermoelectric, piezoelectric, hydropower, etc. Even though it is considered a green renewable energy source, hydropower engineering requires a continuous water source and materials with good hydrophilicity, like graphene, carbon nanotubes, carbon black, etc. [12,13,14,15,16]. Meanwhile, hydropower sensors have limited potential in sewage detection, seawater purification, sweat detection, etc. Photoelectric materials have been widely used in flexible imaging, bionic vision systems, human–machine interactions, etc. [17,18,19]. However, low radiation absorption, high detection limits, and low resolution are still the bottlenecks encountered in the application of photoelectric sensors in accurate detection. The strain-dependent charge output of piezoelectric materials has been typically used for the collection of energy from human movement [20,21,22] and monitoring of eye fatigue [23,24]. Although the previously mentioned methods can yield a beneficial quantity of energy, the focus on thermoelectric materials has intensified owing to their broad prospects in sensing and energy capture applications, along with their capability to transform heat directly into electrical power without the requirement for movable components or upkeep. Given that the human body persistently produces thermal energy within a spectrum of 100 to 525 W, harnessing this wasted body heat through thermoelectric gadgets could result in the generation of 1 to 5.25 W of electrical energy [25].

When the wearable thermoelectric device is attached to the human body, the contacted skin can be regarded as a constant heat source. With the relatively low temperature difference between the skin and atmosphere, thermoelectric devices convert this low-grade heat into electricity, which can power WEDs or work as a temperature sensor. For example, thermoelectric materials can be used as sensors with high measurement accuracy, which can sense subtle changes in the human body and output effective information. Chen et al. reported a large strain hydrogel thermoelectric device that can continuously and spontaneously supply power and monitor human movement at ambient temperature [26]. Liu et al. prepared flexible thermoelectric composite fabrics by electrospinning polylactic acid with carbon nanotubes (CNTs). The device has excellent durability and thermoelectric conversion efficiency [27].

Moreover, thermoelectric devices are able to avoid low-frequency noise, which is common in traditional generators and may cause chronic damage to the human brain. Due to this unique feature, thermoelectric devices are a potential candidate for employing in the medication field. Arakawa et al. reported a Bi_2_Te_3_-based thermoelectric cooling/heating knife that enables rapid changes in tip temperature [28]. Since this thermoelectric knife does not make any noise when raising and lowering the temperature, it can effectively protect the user while cutting objects or in surgery. With the increase in the number of implantable devices for lifesaving therapies and diagnosis, thermoelectric generators have been targeted as the alternative power source when the batteries run out and need to be replaced by surgical operation. Since there is a natural temperature difference between the inner skin and the interior of the body, thermoelectric energy harvesting is an available and long-term power supply for implantable devices [29].

Thermoelectric materials are categorized into classic inorganic semiconductors and organic conductive polymers. Inorganic semiconductors generally exhibit higher thermoelectric performance than organic polymers. Although most of the inorganic thermoelectric materials yield thermoelectric figure of merit (ZT) values higher than 1, their applications are limited due to their inherent shortcomings, including their high cost of manufacturing, heavy weight, rarity, brittleness, and toxicity. Conversely, organic materials are promising for wearable thermoelectric devices due to their characteristics of cost saving, low density, mechanical flexibility, convenient processing, and low thermal conductivity. Organic thermoelectric generators (OTEGs) derived from organic thermoelectric materials can be operated at a temperature gradient between room temperature and near 310 K, which matches the normal physiological conditions of the body. This device can effectively convert the human body’s “waste heat” into electricity to power, which is quite suitable for use by wearable applications [30]. In this review, we will introduce the basics of organic thermoelectrics, and discuss the current organic thermoelectric materials and composites that are used in WEDs. Device assembly strategies are also summarized and discussed. We expect this review will not only serve as a guideline for junior researchers in the field of organic thermoelectrics but also provide a new perspective for potential strategies for WED development.

## 2. Theoretical Principle of Organic Thermoelectrics

### 2.1. Basics of Thermoelectric Effect

**Figure 2:**
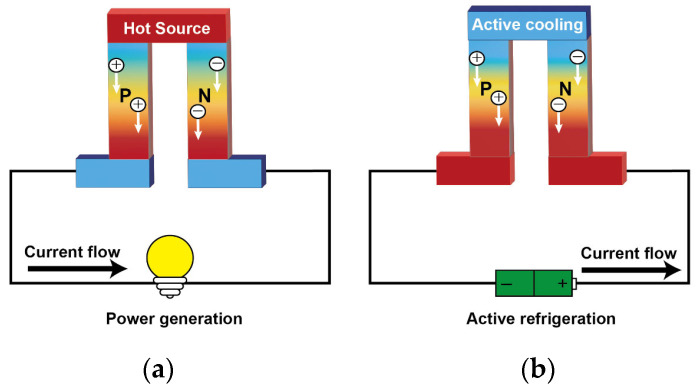
Application of Seebeck and Peltier effects: (**a**) schematic diagram of thermoelectric generator; (**b**) schematic diagram of thermoelectric cooling.

The thermoelectric phenomenon relates a temperature gradient and an electric potential difference across any material, which was discovered in 1822 by Thomas Seebeck. The thermoelectricity refers to three effects: the Seebeck effect, Peltier effect, and Thomson effect [25,31,32]. Figure 2 shows two common thermoelectric phenomena.

The Seebeck effect [33,34,35] is the conversion of temperature gradients to an electrical voltage. It states that, in a circuit formed by two different metals, when a temperature difference is applied, a potential difference (voltage) is generated between the two junctions. If there is load in the circuit, an electric current flow is established. The relationship between the above items is given by the equation

(1) $$V_{ab}=S_{ab}(T_{h}-T_{c})$$

where *V_ab_* is the generated thermoelectric voltage, *S_ab_* is the relative Seebeck coefficient, and *T_h_* and *T_c_* are the temperature of the hot and cold junctions, respectively.

The Peltier effect [36,37,38] can also be regarded as the reverse process of the Seebeck effect: when a current passes through a circuit composed of different conductors, besides the Joule heat, heat absorption and heat release will occur, respectively, at the junction of two different conductors/semiconductors with different electrical current directions. Assuming that the direction of the current is from A to B, the heat absorbed or emitted at the joint is proportional to the current intensity passing between the joints in one unit of time, which can be given as

(2) $$Q_{p}=\Pi_{ab}I$$

where *Π_ab_* is the relative Peltier coefficient and *I* is the electric current passing between the junctions.

The Peltier effect is mainly caused by the difference in Fermi levels between two materials. The movement of charge from a high to low level releases energy in the form of heat in the joints between the two materials, while the movement from a low to high level is the opposite, so the Peltier effect can be used for heating and cooling, depending on the direction of the current flow.

The Thomson effect [39,40,41] occurs if there is a temperature gradient in a uniform conductor; in addition to generating irreversible Joule heat, the conductor also absorbs or emits a certain amount of heat. The relationship can be expressed as

(3) $$Q_{T}=\beta I∆T$$

where *β* is the Thomson coefficient, *I* is the current, and Δ*T* is the temperature difference at junctions between the two materials.

The relationship among the Seebeck coefficient, Peltier coefficient and Thomson coefficient can be clearly expressed by the Kelvin equation:

(4) Π=ST

(5) β=TdS/dT

Generally, the energy conversion efficiency of thermoelectric materials is determined by the dimensionless figure of merit (*ZT*) [42] as follows:

(6) $$ZT=\frac{S^{2}\sigma T}{\kappa }$$

where *S* is the Seebeck coefficient, *σ* is the electrical conductivity, *T* is the absolute temperature, and *κ* is the thermal conductivity.

To achieve a high energy conversion efficiency, the materials should possess a large Seebeck coefficient, high electrical conductivity, and low thermal conductivity [43]. However, these properties are highly interdependent, so changing one property may result in adverse changes in the other two properties.

Electrical conductivity (*σ*) is the quantification of carrier movement in conductive or semi-conductive materials, which can be expressed as

(7) $$\sigma =e(\mu_{e}n+\mu_{h}p)$$

where *e* represents the unit charge; *n* and *μ_e_* are the concentration and mobility of electrons; and *p* and *μ_h_* are the concentration and mobility of holes.

The Seebeck coefficient is defined as the entropy change caused by thermally excited carrier transport because the carriers move under the temperature field. It describes the material property to generate an open-circuit voltage when submitted to a given temperature gradient:

(8) $$S=\frac{V_{oc}}{\Delta T}$$

where *V_oc_* is the open-circuit voltage and Δ*T* is the temperature difference. In homogeneous materials, the Seebeck coefficient is affected by the Fermi level (*E_F_*), state density, carrier concentration, and other factors. 

The carrier density is the key factor affecting the Seebeck coefficient and the conductivity level of materials, which are generally related to the thermoelectric properties of the material [44]. Therefore, the measurement of carrier density is necessary to optimize the thermoelectric properties of materials, but it is not easy to quantitatively verify the relationship between the carrier density and Seebeck coefficient, mainly because it is not easy to change the charge density in thermoelectric materials, and it is not easy to accurately evaluate its value. Imae et al. used PSC (potential-step chronocoulometry) to measure the doping level of P3HT synthesized by different polymerization methods, thus establishing a correlation with thermoelectric properties, and finally proved that the saturation value of the doping level of the P3HT under different polymerization methods was 25% [45]. That is, about one positive charge is injected into every four thiophene rings (one bipolar ring for every eight thiophene rings). Of course, for the optimization of thermoelectric materials, it will ultimately depend on the ZT value, which is the most critical factor in measuring the thermoelectric performance of materials. The peak value of ZT generally occurs in the carrier concentration range of 10^19^–10^21^ cm^−3^ [46]. However, the intrinsic carrier concentration of most organic thermoelectric materials is much lower than this value, so researchers have developed a variety of dopants and doping methods to regulate the carrier concentration and electron energy level structure, so as to improve the thermoelectric value.

Similar to charge transport, the thermal conductivity of a material depends on the amount of heat carried and transported during the movement of charge carriers and phonons, i.e.,

(9) $$\kappa =\kappa_{e}+\kappa_{l}$$

where *κ* is the total thermal conductivity of the material, *κ_e_* is the thermal conductivity contributed by the carrier, and *κ_l_* is the thermal conductivity contributed by the phonon. At present, the reported thermal conductivity of organic material films is 0.1–1 W m^−1^·K^−1^ [47,48,49,50]. In organic materials, because the molecular regularity of organic materials is worse than that of inorganic materials, it is difficult to form a lattice structure and the conductivity is low, so the thermal conductivity of electrons brought by carrier transport is generally low; that is, the thermal conductivity of electrons is far less than the thermal conductivity of phonons [51,52]. The orientation of the filler, the structure of the material, the arrangement of molecular chains, lattice defects, and other factors will affect the thermal conductivity, and with a change of temperature, the thermal conductivity may also change. 

### 2.2. Recently Studied Organic Thermoelectric Materials

**Figure 3:**
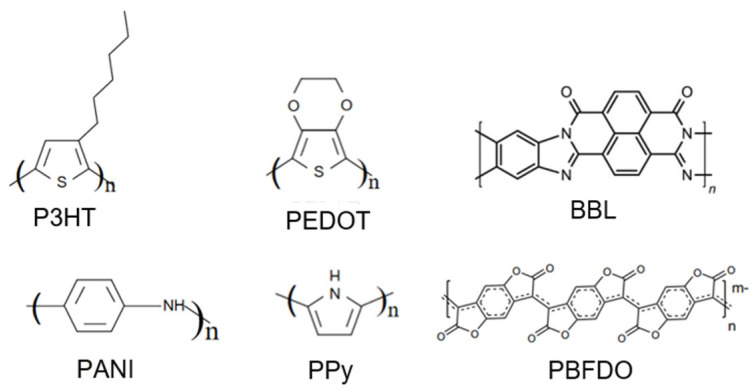
Molecular structures of organic thermoelectric materials.

Conductive polymers are widely used as organic thermoelectric materials; these include poly(3,4-ethylenedioxythiophene) (PEDOT), polyaniline (PANI), polypyrrole (PPy), poly(3-hexylthiophene) (P3HT), and some n-type conducting polymers such as poly(benzodifurandione) (PBFDO) and poly(benzimidazobenzophenanthroline) (BBL). Some of these molecules’ structures are shown in Figure 3. In this section, we will briefly discuss the representative organic thermoelectric materials.

#### 2.2.1. PEDOT

As a derivative of polythiophene, PEDOT has been wildly investigated since 1988 by the Bayer AG laboratory in Germany. This process involves the oxidative polymerization of EDOT monomers, which gives rise to oligomers characterized by a low ionization potential and a tendency to undergo spontaneous oxidation in the presence of an oxidant. The PEDOT polymer chains that result are usually p-doped with negative counterions to maintain electrical neutrality. Commonly utilized counterions include tosylate ions (Tos-) and the poly(styrene sulfonate) ions (PSS-), leading to the formation of PEDOT:Tos and PEDOT:PSS. Unlike in inorganic materials, ‘doping’ in organic materials refers to oxidizing or reducing the conjugated polymers and increasing the carrier density. One of the advantages of organic conductive materials is that they can be further chemically or electrochemically treated to improve their electrical conductivity. 

**Figure 4:**
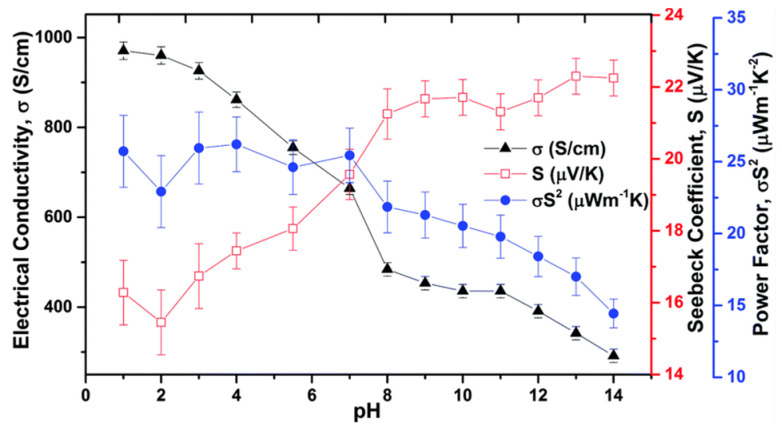
Effect of pH on Seebeck coefficient, conductivity, and power factor of PEDOT:Tos films [53].

In research conducted by Crispin et al. in 2011, PEDOT:Tos films were synthesized through two distinct techniques: vacuum vapor phase polymerization and chemical polymerization [53]. The researchers embarked on fine-tuning the thermoelectric properties of these films, investigating the influence of different pH levels. The observed discrepancies in the Seebeck coefficient and electrical conductivity with pH were primarily attributed to alterations in the oxidation state of PEDOT. According to the data in Figure 4, the films produced via vacuum vapor phase polymerization exhibited a reduction in electrical conductivity from approximately 970 S cm^−1^ to 290 S cm^−1^, while the Seebeck coefficient rose from 15 µV K^−1^ to 23 µV K^−1^ as the samples transitioned from pH 1 to pH 14 solutions. The authors proposed that this effect might be due to the replacement of tosylate ions with Cl- or OH- ions, which would alter the spacing and π-stacking within the film’s molecular structure. Ptsagkourakis and colleagues examined the thermoelectric performance of various PEDOT:Tos films, identifying a relationship where the Seebeck coefficient is proportional to the charge carrier mobility to the power of 0.2, an observation that had not previously been reported in the field of organic electronics [54]. As the crystallinity of the films incrementally increased, the material evolved from a Fermi glass into a semi-metal state, paving the way for a highly efficient network for charge transport. Their findings underscored the critical role of improved carrier mobility in crafting high-performance thermoelectric polymers. 

Pure PEDOT is not soluble in water. However, the unique water processability of PEDOT:PSS is attributed to the sulfonate groups on the PSS, which allows for aqueous dispersion. However, the electric conductivity of PEDOT:PSS films at around 10^−2^ S cm^−1^ is still insufficient to meet the demands for many applications. Enhanced performance can be realized by manipulating the film’s structure and polymer alignment through the use of high-boiling-point solvents or acidic treatments. 

Kim et al. studied the effects of various organic solvents on the electrical conductivity of PEDOT:PSS films [55]. Three solvents, dimethyl sulfoxide (DMSO), N, N-dimethylformamide (DMF), and tetrahydrofuran (THF), were mixed with a PEDOT:PSS aqueous solution in a certain proportion, and then cast to form free-standing films. XRD, XPS, and EPR (electron paramagnetic resonance) characterization confirmed the same polymer chain conformation and doping level of the different systems. They predicted that the screening effect induced by the polar solvent was a vital contributing factor for the charge transport properties and temperature dependence. Jiang et al. improved conductivity by incorporating exfoliated MoS_2_ nanosheets into the PEDOT:PSS solution, using common organic solvents and vacuum filtration to fabricate thin films. This method not only enhanced the conductivity (up to 1250 S cm^−1^) by partially removing PSS but also slightly raised the Seebeck coefficient from 14.5 to 19.5 μV K^−1^ [56]. 

Kim et al.’s approach to boosting the thermoelectric performance involved exposing PEDOT:PSS films to sulfuric acid vapor. This weakened the Coulomb interaction between the PEDOT and PSS chains and changed the film morphology to expanded coil-like nanostructures [57]. Post treatment with H_2_SO_4_ vapor at 140 °C drastically improved the film’s conductivity from 1.1 to 1167 S cm^−1^, and the Seebeck coefficient increased from 7.3 to 12.1 μV K^−1^. Meanwhile, the maximum value of the power factor reached 17.0 μW m^−1^ K^−2^, which is a significant jump from that of the untreated film (0.006 μW m^−1^ K^−2^). 

**Figure 5:**
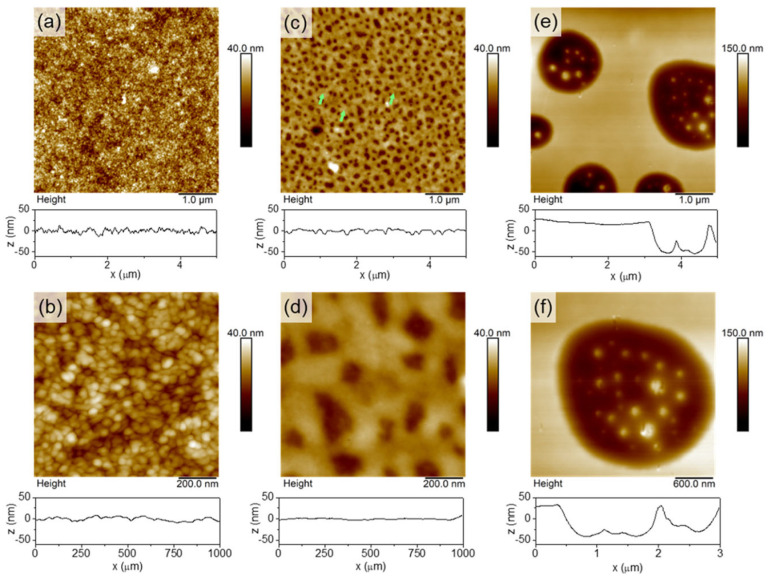
AFM topography of THF-treated PEDOT:PSS films. (**a**,**b**) pristine sample, (**c**,**d**) after short interaction times (te ≪ 1 min), and (**e**,**f**) after long interaction times (te = 10 min). A topography cross-section, taken at the center of each image, is presented below each ffgure [69].

Recently, researchers have begun to develop ionic liquids for use as dopants. Saxena et al. explored the use of ionic liquids as dopants for PEDOT:PSS films, treating them with 1-ethyl-3-methyl imidazolium dicyanamide (EMIM:DCA), 1-ethyl-3-methyl imidazoliumtetracyanoborate (EMIM:TCB), and 1-ethyl-3-methyl imidazolium tetrafluoroborate (EMIM:BF_4_) in THF [58]. The research indicated that the varying Seebeck coefficients were linked to the ionic liquids’ anionic chemical properties. Imae et al. further explored the effect of ionic liquids (EMIM:TCB) with different concentrations on the thermoelectric properties of PEDOT:PSS films. Studies have shown that hydrophobic and flexible independent films with very high mechanical strength can be easily obtained through EMIM:TCB treatment [59]. Secondly, the concentration of EMIM:TCB affects the crystallinity and carrier density of the films, but has little effect on the Seebeck coefficient and thermal conductivity of the films. Furthermore, the anion types influence the film morphology, such as the average π-π stacking distances between PEDOT chains, the PEDOT to PSS ratios, and edge-on-to-face-on ratios, which collectively affect the macroscopic charge transport properties. Controlling the nanostructure and morphology of PEDOT:PSS is also an important path to regulating its thermoelectric properties. So far, researchers have prepared PEDOT:PSS with different nanostructures, such as linear nanostructures or nanogratings, nanocavities, nanoholes/columns, etc., by laser irradiation [60,61,62,63], nanoimprint lithography [64,65,66], and the template method [67,68]. Sanviti et al. placed PEDOT:PSS films in THF vapor to form different nanostructures depending on the length of the treatment time, which directly affected the thermoelectric properties [69]. Figure 5 shows the AFM images of THF treated PEDOT:PSS films. Through the characterization of the films’ nano-mechanics, nano-electricity, and nano-infrared properties, it was found that the method is simple, fast, and can be selectively modified, and has a very good application prospect. Although the thermoelectric properties of PEDOT films can be effectively improved by adjusting the morphology of the films, the fragility of PEDOT films makes this difficult to apply in practice. In order to solve this issue, Imae et al. explored the use of sulfonated polyhydroxyl ether instead of PSS for doping PEDOT films, and found that polysiloxane-based polyanion doping can improve the flexibility of the films. The higher the molecular weight of the dopant polyanion, the higher the film’s mass and charge mobility [70].

In addition to basic research, the actual production process needs more simple and efficient methods. Zhang et al. mixed commercial PEDOT:PSS aqueous solutions with SWCNT in different proportions to prepare composites. A DMSO treatment afterwards was used to optimize the material properties, and the power factor reached 300 μW m^−1^ K^−2^ under the condition of 74 wt% SWCNT [71]. It is worth noting that Zhang et al. used a high-speed non-solvent turbulent secondary doping (HNTD) method for PEDOT; the properties of the PSS and SWCNT composites were further optimized, and a composite film with a power factor of 501.31 ± 19.23 μW m^−1^ K^−2^ was obtained under the condition of 40 wt% SWCNT, which is the highest among the commercial carbon fillers and organic semiconductor composites [72].

#### 2.2.2. PANI

PANI is another widely studied conductive polymer which was discovered in the 19th century. Its thermoelectric properties and property improvement strategies have been extensively studied in the past few decades. Liu et al. treated PANI with different acids (HCl and toluene-p-sulfonic acid) and measured the changes in thermoelectric properties [73]. The results showed that (1) with the increase in the molecular weight of PANI, its conductivity increased and the Seebeck coefficient decreased, and (2) after reduction using organic acids and HCl, the conductivity and Seebeck coefficient of PANI were increased. Yan et al. alternately assembled (±) -10-camphorsulfonic acid (CSA)-doped PANI and pristine PANI through layer assembly to prepare multilayer PANI films [74]. The test results showed that this method can increase the power factor by 3.5 times to more than 5 μW m^−1^ K^−2^ compared to the single-layer film, and the mechanical properties were correspondingly improved. Then, Qin et al. prepared an SWCNT/PANI composite by in situ polymerization with single-wall carbon nanotubes (SWCNTs) as the dopant [75]. The thermoelectric properties of the composites were significantly improved, with the conductivity increasing up to 1.25 × 10^4^ S cm^−1^ and the Seebeck coefficient increasing up to 40 μV K^−1^. A series of subsequent tests indicated that the molecular structure of the PANI within the composite was more ordered compared to that of the standalone PANI. This increased orderliness is likely due to the robust π–π interactions between the PANI and the nanotubes. The ordered structure resulted in an increase in carrier mobility. Therefore, the electrical conductivity and Seebeck coefficient of the composites were significantly improved. In the case of a high SWCNT content, the thermal conductivity of the composite slightly changed and remained at a very low level, which was caused by the phonon scattering effect at the nanointerface generated by the SWCNT/PANI nanostructure. These findings indicated that chemically stretching the chain structure of conductive polymers represents a new and potent approach to enhance their thermoelectric properties. They then further introduced the self-assembled supramolecule (SAS) 3, 6-dioctyldecoxy-1, 4-phenyl dicarboxylic acid as templates in the preparation of PANI films. The PANI film prepared by this method had a power factor that was 6 times higher with the same thickness. This experimental result demonstrated that the addition of a self-assembling substance prompts an orderly structure and alignment of the PANI molecular chains, a phenomenon that becomes more pronounced as the film thickness diminishes. As a result, the carrier mobility and thermoelectric efficiency of PANI-SAS nanomembranes were significantly improved.

Similar to PEDOT:PSS, PANI can also be used to improve thermoelectric properties through nanotechnology. Wu et al. employed a soft template technique to prepare PANI nanostructures (including nanorods, nanowires, etc.) doped with different acids. Their study discovered that factors like the level of doping, the type of acid used for doping, and the morphology of the nanostructures significantly impacted the thermoelectric performance [76]. For example, PANI nanowires with a high doping concentration of hydrochloric acid did not exhibit superior thermoelectric properties, which was ascribed to the doping of larger anions to make the PANI chain more ordered. When examining the influence of morphology, two samples doped with p-TSA (nanowires and nanorods) showed distinct thermoelectric behaviors despite having the same doping levels. The nanowires demonstrated a higher Seebeck coefficient and lower thermal conductivity compared to the nanorods. Specifically, the Seebeck coefficient of the nanowires was 164% higher, and thermal conductivity was 25% lower. Consequently, the ZT for the nanowires was 5 times higher than that of the nanorods.

#### 2.2.3. BBL

**Figure 6:**
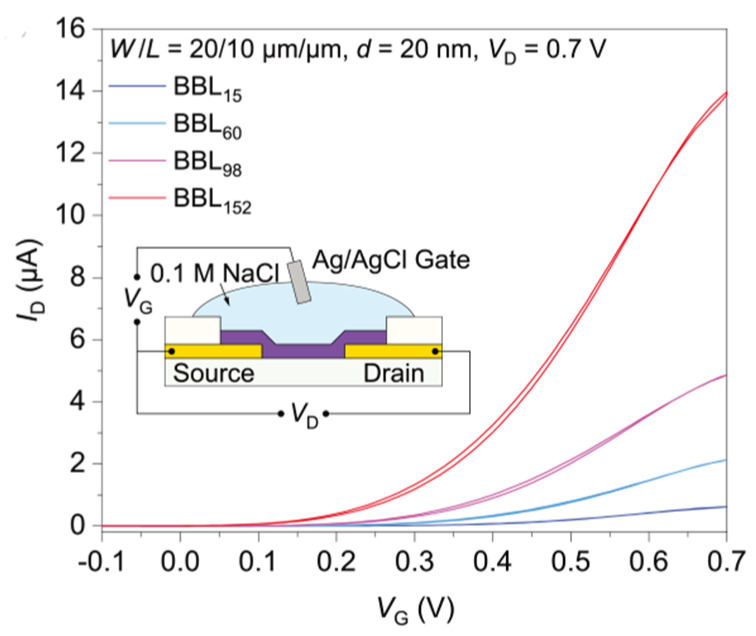
Transfer characteristics of BBL15, BBL60, BBL98, and BBL152-based OECTs (Voltammetry curves of BBL with different molecular weights). Inset: cross-sectional schematic of the OECTs [77].

P-type conductive polymers were the most studied organic thermoelectric materials for a long time because of its relatively high performance in comparison with the n-type polymer. BBL was one of the earliest studied n-type conductive polymers with a fair conductivity. Wu et al. prepared BBL with different molecular weights (from 4.9 KDa to 51 KDa) by controlling the polymerization reaction time, and studied the relationship between the BBL molecular weight and electrical properties [77]. Figure 6 shows the transfer characteristics of different BBL based OECTs. The experimental results showed that with an increase in molecular weight, the effective conjugation length of BBL increased, the π-π packing distance decreased, the crystallinity of BBL was enhanced, and the electrochemical doping potential decreased, which could be conducive to electron transport. The experiment also demonstrated that the molecular arrangement of the fully rigid trapezoidal copolymer was less affected by molecular entanglement, so increasing the molecular weight can significantly improve the electrical properties of the polymer.

Secondly, by adjusting the structure or molecular conformation of the BBL molecular chain, its performance can also be significantly improved. He et al. reported the preparation and characterization of a π-expanded polybenzimidazolantrone isoquinolinedione (BAL) [78]. Tetrachloride BAL (Cl_4_-BAL) is completely soluble in methyl sulfonic acid and can be spun into high-quality films, making the preparation and characterization of organic thermoelectric possible. Doping Cl_4_-BAL films with phenylviolet cations (BV●+) resulted in better electro-air stability compared with BBL owing to its extremely low LUMO (lowest unoccupied molecular orbital) value (−4.83 eV).

It is worth noting that when BBL is highly doped, the Seebeck coefficient of BBL may be reversed. Fabiano et al. doped BBL using the electrochemical doping method and studied the evolution of the Seebeck coefficient under different doping levels [79]. The results showed that when 0.7 electron/repeating units were doped, the electrical conductivity reached a peak value of 3.1 S cm^−1^, while at higher doping levels, the conductivity decreased to almost a non-conductive state. Advanced techniques such as in situ EPR, UV–vis–NIR spectroscopy, and Raman spectro-electrochemistry, along with DFT/TD-DFT calculations, confirmed that high doping levels resulted in the formation of multi-charged entities with poor mobility. Further exploring the charge transport in BBL, the researchers measured the Seebeck coefficient at various charge densities and discovered a transition from n-type to p-type behavior in the organic semiconductor. The data from Kinetic Monte Carlo simulations were consistent with the experimental results, providing evidence that the shift in the Seebeck coefficient was due to the filling of the density of states and the emergence of a hard Coulomb gap near the Fermi energy.

#### 2.2.4. PBFDO

**Figure 7:**
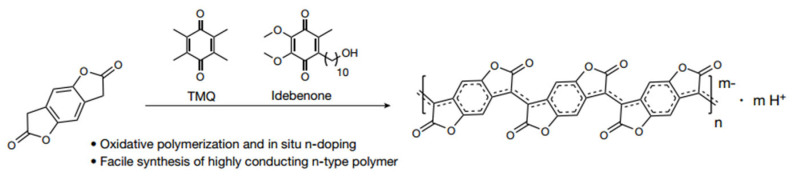
Synthesis and structural formula of PBFDO [80].

As an emerging n-type organic material, PBFDO exhibits excellent thermoelectric properties, solution conductivity exceeding 2000 S cm^−1^, and excellent stability and solution processability. Huang et al. applied oxidative polymerization and in situ reductive n-doping to significantly improve the doping efficiency (the doping level of each repeating unit was close to 0.9 charges) and prepared a rigid conjugate skeleton with a high doping level, as shown in Figure 7 [80]. It is worth noting that the polymer had no side chains, which was convenient for further operations such as side chain modification.

Subsequently, they further studied the effect of the interaction between electrolytes and PBFDO on the performance of organic electrochemical transistors (OECTs) [81], and found that PBFDO’s electronic band structure was slightly affected by the increased ion concentration after doping; electrolytes with a high salt concentration were more effective at speeding up the electrochemical reactions. In addition, compared to the concentrated solutions, diluted solutions significantly increased the film’s surface roughness and reduced the crystal coherence length. A quantitative analysis using electrochemical quartz crystal microbalances showed that the electrolyte ions penetrated the PBFDO membrane, inducing water molecule absorption, a process that was pronounced in diluted solutions and negligible in concentrated ones. This swelling of the polymer in dilute solutions could potentially obstruct charge carrier transport, thereby affecting OECT performance. Table 1 summarizes the thermoelectric properties of some common organic thermoelectric materials [80,82,83,84,85,86,87,88,89,90,91,92,93,94,95,96,97]. 

## 3. Types of Organic Thermoelectric Materials

According to the material morphology, the types of organic thermoelectric materials applied to WEDs can be mainly classified as thermoelectric fibers, thermoelectric films, and bulk thermoelectric materials.

### 3.1. Organic Thermoelectric Fibers

For wearable electronic devices (WEDs), fiber-based thermoelectric materials are gaining extensive interest due to their lightweight, flexible, and deformable properties. Wen et al. developed high-performance PEDOT:PSS thermoelectric fibers through a continuous wet-spinning process, followed by a one-step H_2_SO_4_ treatment [98]. The fibers exhibit a power factor of 147.8 μW m^−1^ K^−2^, an electrical conductivity 4029.5 S cm^−1^, and a Seebeck coefficient of 19.2 μV K^−1^. The power factor was 15 times higher than that of an untreated PEDOT:PSS film prepared under the same conditions. Meanwhile, the PEDOT:PSS fibers possessed a high tensile strength of 389.5 MPa and a large breaking strain of 30.5%.

Chen et al. increased the thermoelectric properties of PEDOT:PSS fibers by adding an ionic liquid (EMIM:DCA) to obtain an electrical conductivity higher than 4000 S cm^−1^ [99]. The results of the SEM, XPS, Raman, UV–vis–NIR, and wide-angle X-ray scattering (WAXS) analyses revealed that the improved thermoelectric performance was due to the substantial removal of PSS, a high crystallinity degree (87.9%), and a high orientation (0.71) of the PEDOT molecules. This improvement was ascribed to the synergistic effects of the ionic liquid, concentrated H_2_SO_4_, and intense stretching. 

**Figure 8:**
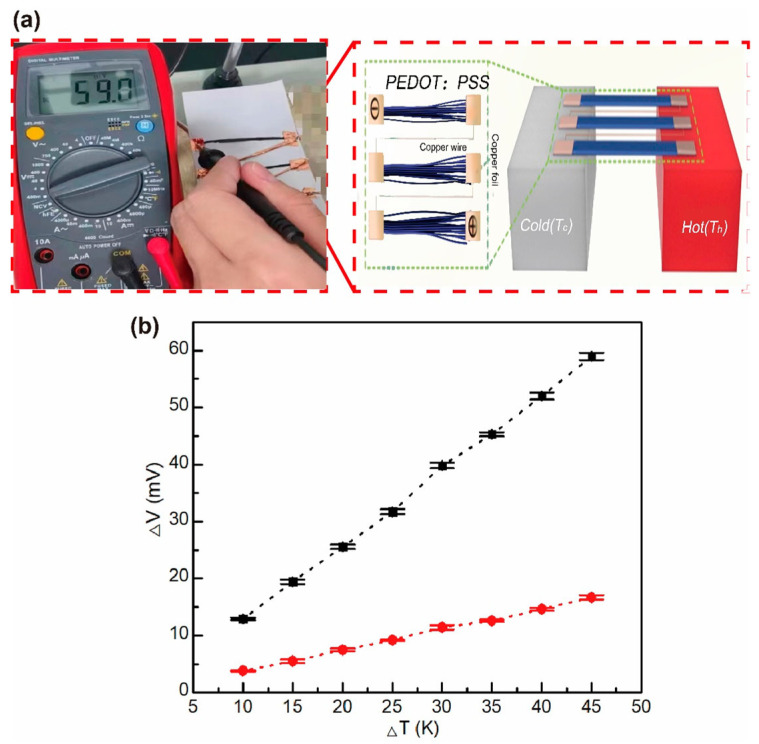
(**a**) Thermoelectric generator; (**b**) output voltages for different thermoelectric devices [100].

Xiao et al. also created PEDOT:PSS fibers with high thermoelectric performance by low-temperature in situ polymerization, freeze–thaw treatment, and the wet-spinning method, as shown in Figure 8 [100]. By fine-tuning the polymerization reaction temperature and the number of freeze–thaw cycles, they significantly enhanced the fibers’ properties. The optimized PEDOT:PSS fibers, produced at a low in situ polymerization temperature (−18 °C) and after two freeze–thaw cycles, achieved a high Seebeck coefficient of 40.8 μV K^−1^, an electrical conductivity of 980 S cm^−1^, and a tensile breaking strength of 57.42 cN.

**Figure 9:**
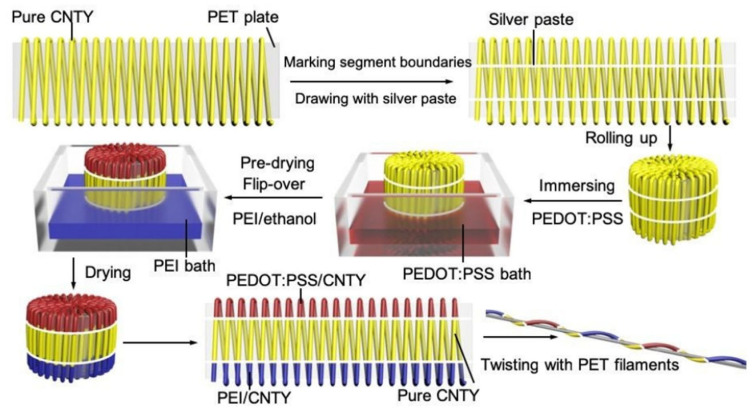
Schematic diagram of the preparation process of thermoelectric yarns [101].

Zheng et al. reported a strategy for the efficient preparation of fiber/yarn-based thermoelectric textiles with a high output power, and the three-dimensional textiles also showed quite good flexibility under bending, twisting and compression, as shown in Figure 9 [101]. CNT yarns were subsequently dipped into an aqueous PEDOT:PSS solution and PEI/ethanol solution to form the periodic units of p-legs and n-legs. Thermoelectric textiles were then obtained by knitting the thermoelectric yarns into the weft-knitted spacer fabric. Under the condition of ΔT = 47.5 K, the output power density of the thermoelectric textile was higher than 50 mW m^−1^ K^−2^, and the specific power density was as high as 171.7 μW g^−1^ K^−1^.

Li et al. fabricated novel flexible CNT/PANI fibers with high thermoelectric performance using the wet-spinning approach [102]. They modulated the CNT content and fine-tuned the fabrication conditions, such as the time in the coagulation bath, needle diameter, extrusion rate, and doping level of the fibers. The highest value of PF was demonstrated to be 176 μW m^−1^ K^−2^ and was superior to that of most of the previously reported conducting polymer-based fibers. 

### 3.2. Organic Thermoelectric Films 

**Figure 10:**
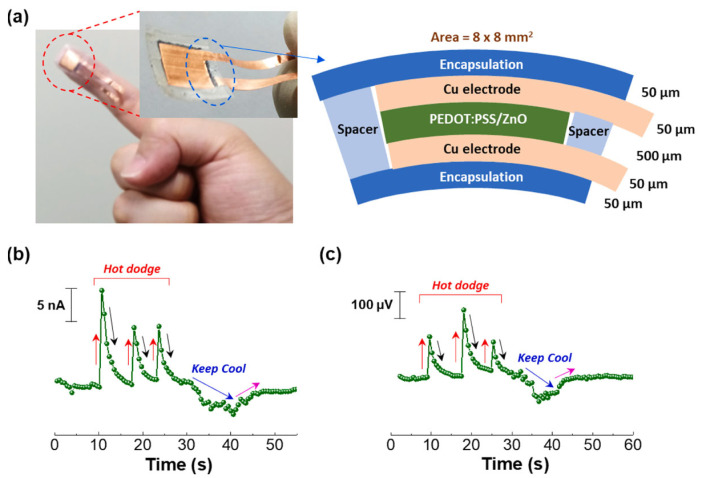
(**a**) A fingertip-mounted flexible thermal sensor based on PEDOT:PSS/ZnO hybrid composite sheets (ZnO content = 70 wt%) (right: cross-sectional structure). (**b**) Current and (**c**) voltage responses of the flexible thermal sensor as it is moved towards heat and cold sources (red arrow: near heat source; black arrow: moving away from heat source; blue arrow: close to the cold source; pink arrow: moving from cold source) [103].

Thin films have been widely studied because of their simple preparation process. Interactions between the sulfonic acid groups in PSS and the hydroxyl groups (and/or zinc cations) in ZnO cause an increase in the viscosity of PEDOT:PSS aqueous solutions upon ZnO particle integration. This leads to improved thermoelectric properties in ZnO/PEDOT:PSS composites when compared to unmodified PEDOT:PSS. Lee and colleagues examined the morphological and compositional nuances of PEDOT:PSS composite films containing ZnO particles of varying sizes (0.1, 5, and 45 μm) [103]. It was found that composites with a 70 wt% concentration of 45 μm ZnO particles achieved a power factor of 1.7 × 10^−6^ μW m^−1^ K^−2^ at ΔT = 50 K. Furthermore, wearable thermal sensors using these PEDOT:PSS/ZnO composite films demonstrated rapid thermoelectric responses to heat and cooling sources, as depicted in Figure 10 [103]. 

Liu et al. prepared PEDOT:PSS/Cu_2_S films using the vacuum filtration method and studied the effects of the Cu_2_S content and the pressure from cold-pressing on the thermoelectric properties of composite films [104]. An increase in the Cu_2_S content resulted in reduced electrical conductivity, while the Seebeck coefficient increased. The films with a 10% Cu_2_S content at 393 K achieved the highest power factor of 56.15 μW m^−1^ K^−2^. Interestingly, cold-pressing at 2 MPa or 4 MPa enhanced the films’ power factor. A thermoelectric generator composed of four cold-pressed strips of 10 wt% PEDOT:PSS/Cu_2_S composite films showed potential for powering low-energy consumption devices with an open-circuit voltage of 2.3 mV and maximum output power of 23.06 nW at a 30 K temperature difference.

Paulraj et al. investigated the synergistic impact of ethylene glycol and reducing agents (EG/NaBH_4_ or EG/NaHCO_3_) on the thermoelectric properties of PEDOT:PSS films [105]. According to the Hall measurements and thermoelectric test, the charge carrier concentration and Seebeck coefficient changed markedly, which was attributed to the variation in the density of states at the Fermi level. After treatment with a 0.05 MEG/NaHCO_3_ solution, the film exhibited the maximum power factor of 183 μW m^−1^ K^−2^ and a Seebeck coefficient of 48 μV K^−1^ at 450 K. For the device made from four pairs of p-legs (EG/NaHCO_3_-treated PEDOT:PSS) and n-legs (Cu_0_._6_Ni_0_._4_) with dimensions of 4 mm × 20 mm for each leg on the polyamide substrate, the corresponding maximum power density achieved was 98 μW cm^−2^.

**Figure 11:**
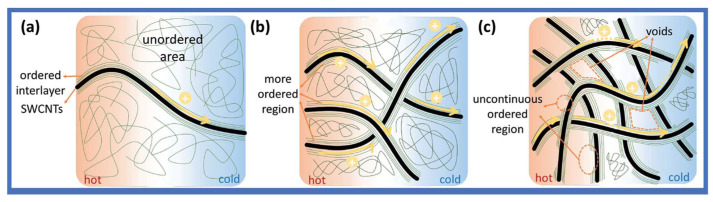
Schematic diagram of electron transport in composite films: (**a**) SWCNTs < 10 wt%, (**b**) SWCNTs > 10 wt%, and (**c**) SWCNTs > 90 wt% [106].

PANI films have been widely studied for their high conductivity and good stability. Wu et al. systematically investigated the effect of PANI with different polymerization times on the thermoelectric properties of SWCNT/PANI composite films [106]. The results showed that with an increase in polymerization time, the mechanical properties of the film correspondingly improved, and a super high electrical conductivity (~4000 S cm^−1^) was obtained under the optimal conditions. The investigation indicated that the cohesive and systematic interfacial regions bridging the filler and the matrix were pivotal in enhancing the electrical conductivity of the composite films. It is noteworthy that the Seebeck coefficient exhibited a consistent and marginal increase as the SWCNT content increased across the entire range examined. The power factor ultimately attained a peak value of 100 μW m^−1^ K^−2^. Figure 11 shows the different electron transport mechanisms of varies SWCNTs/PANI composites.

Zhu et al. reported a bottom-up preparation method for the synthesis of molecular-level polymer/nanoparticle hybrids, effectively scaling the process for device-level applications and thus linking nanoscale material interactions to macroscopic energy conversion efficiencies [107]. The interface between the PANI polymer chains and the multi-walled carbon nanotubes (MWCNTs) was optimized by in situ polymerization, which improved the power factor (to ~1.25 μW m^−1^ K^−2^) when using a high MWCNT concentration of 58.7 wt%. The resultant flexible thermoelectric generator, combining PANI/MWNT hybrid films with a thermoplastic polyurethane (TPU) substrate, demonstrated its utility by powering a sensory system that could detect and monitor long-term bio-signals, including joint movements and respiration.

**Figure 12:**
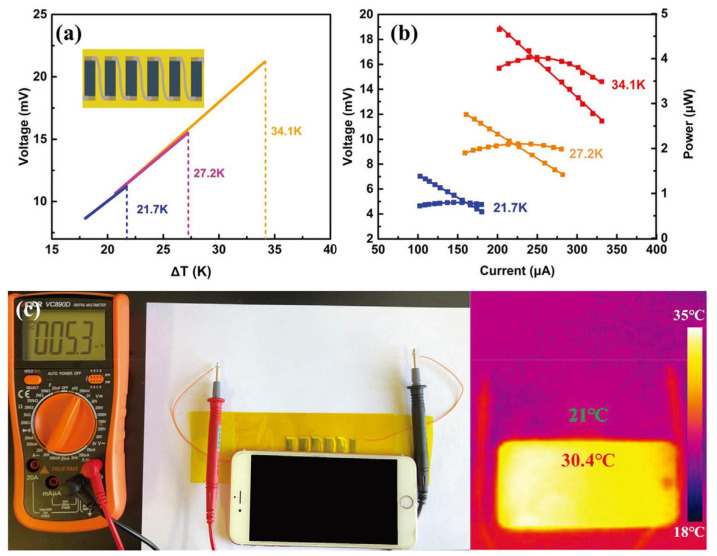
Properties of six-leg thermoelectric generator (TEG) manufactured with the film. (**a**) Open-circuit voltage at different ΔT values (illustrated as schematic diagram of f-TEG). (**b**) The measured voltage and output power at different applied currents and ΔT values. (**c**) Photo of 5.3 mV voltage generated by ΔT between the just-used mobile phone and the surrounding environment (corresponding infrared thermal image on the right) [108].

Li et al. developed Ag_2_Se nanostructures utilizing a wet chemical synthesis method, polymerized PPy directly onto the surface of these nanostructures, and ultimately engineered a Ag_2_Se/Se/PPy composite film atop a porous nylon membrane using vacuum-assisted hot pressing [108]. This film exhibited an exceptional power factor of approximately 2240 μW m^−1^ K^−2^ at 300 K, which was attributed to the synergistic influence of crystalline Ag_2_Se grains alongside minute quantities of Se and PPy. In addition, a six-leg flexible thermoelectric generator could provide a voltage of 21.2 mV and a maximum power output of 4.04 µW (power density of 37.6 W m^−2^) at ΔT = 34.1 K, which was quite hopeful for its use in practical applications in wearable devices, as shown in Figure 12.

### 3.3. Bulk Organic Thermoelectric Materials

The most common bulk organic thermoelectric materials include aerogels, hydrogels, etc. Organic aerogels are a promising candidate for TEG applications because of their low thermal conductivity and superior elasticity. As components of aerogels, nature-derived polymeric materials have gained attention owing to their abundant availability, easy preparation, and environmental friendliness. 

Su et al. reported an aerogel with thermoelectric properties. This aerogel was assembled based on carboxylated nanocellulose fibers and CNTs using directional freezing [109]. The aerogel had excellent mechanical properties thanks to the presence of cellulose. In addition, it also had low thermal conductivity and a low density. 

**Figure 13:**
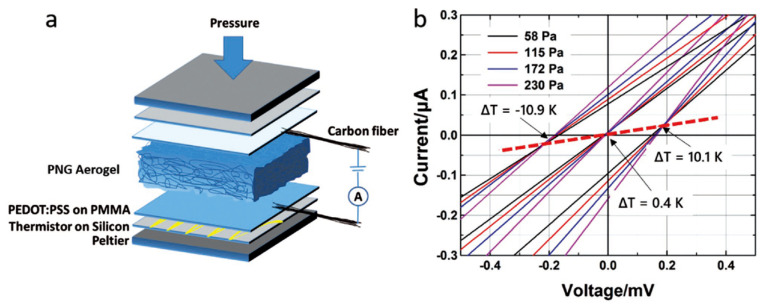
(**a**) Schematic diagram of a sensor device prepared with PNG aerogel. (**b**) Current−voltage curves measured at different temperatures and pressures [110].

Han et al. reported thermoelectric polymer aerogels which were fabricated by a freeze drying strategy with three organic materials: a conductive polymer (PEDOT:PSS), skeleton material (NFC), and cross-linker (GOPS); these aerogels are defined as PNG aerogels (Figure 13) [110]. The carrier transport properties of those conductive aerogels were subsequently fine-tuned using the vapor of the high-boiling-point polar solvent DMSO. The resistance of the PNG aerogel was markedly reduced from approximately 2 kΩ to 44 Ω, consequently leading to a profound augmentation in its pressure sensitivity from 3 × 10^−7^ to an impressive 2 × 10^−5^ A Pa^−1^ (measured at 100 mV) after treatment with DMSO vapor.

**Figure 14:**
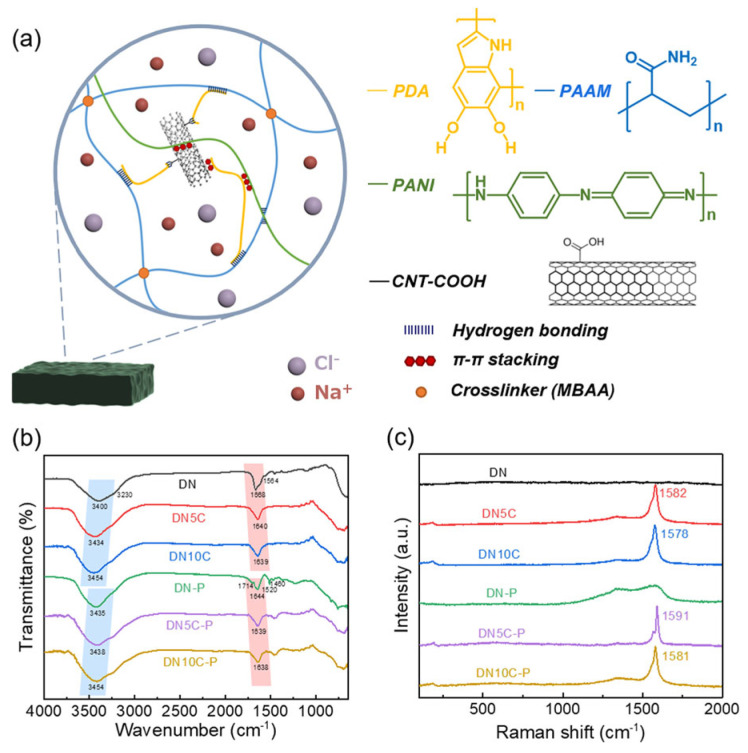
(**a**) Structure of PDA/CNT/PAAM/PANI hydrogels with four different chemical structures. (**b**) FTIR and (**c**) Raman spectroscopy of the hydrogels [111].

Lee and colleagues prepared a dual-network hydrogel by chemically cross-linking polyacrylamide (PAAM) and polydopamine (PDA) and subsequently dispersed carboxylated carbon nanotubes (CNT-COOHs) through a combination of ultrasonication and interaction with the PDA matrix, as shown in Figure 14 [111]. The authors then proceeded to amplify the thermoelectric capabilities of this hydrogel through in situ polymerization of PANI. The X-ray photoelectron spectroscopy analyses revealed the pivotal role of the CNT-COOHs and the ionic–electronic interactions between sodium ions and the carboxyl moieties of the CNT-COOHs in facilitating charge transport. The hydrogel exhibited an ionic conductivity of 175.3 S cm^−1^, a Seebeck coefficient of 18.6 μV K^−1^, and an ionic power factor above 6 μW m^−1^ K^−2^, culminating in an impressive ionic figure of merit (ZTi) of 2.65. A wearable thermoelectric module, prepared by encapsulating the PAAM/PDA/CNT-COOH/PANI hydrogel within a poly(dimethylsiloxane) scaffold, demonstrated a remarkable power density of 171.4 mW m^−2^.

On another frontier, He et al. reported the development of a multifaceted phase-change organic hydrogel that encapsulates paraffin wax (PW) microspheres utilizing the Pickering emulsion methodology coupled with UV-initiated polymerization [112]. The innovative strategy of confining PW within a robust composite shell composed of cellulose nanofibril (CNF), MXene, and Fe_3_O_4_, and then embedding it within a flexible polyacrylamide (PAAm) matrix improved the thermoelectric conversion efficiency, form stability, and mechanical integrity. The thermoelectric generator output a voltage of 627.0 mV and a power of 65.7 mW. Moreover, due to the satisfactory thermosensing capacity, the composite organohydrogel could find application in the real-time monitoring of temperature fluctuations and the behavior of hot steam.

**Figure 15:**
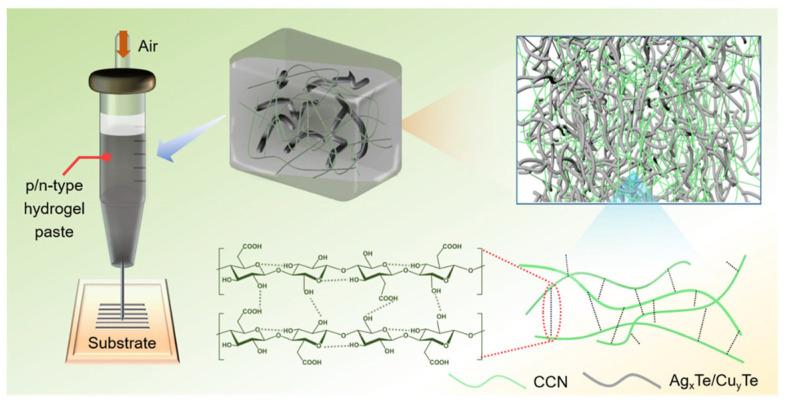
The mechanism of physical crosslinking, the relationship between the AgxTe/CuyTe NR dispersion constraint and high aspect ratio CCN hydrogel pastes, and their application in printing ink [113].

Confronting the challenge of diminished electrical conductivity in the fabrication of flexible thermoelectric devices via printing methods, Wu’s team put forth a hydrogel-based printing technique for the deposition of flexible thermoelectric generators on a plethora of substrates, as shown in Figure 15 [113]. The robust hydrogel network, constituted by carboxylated cellulose nanofibers, adeptly limited the fluidity of the 1D nanorod dispersion, resulting in a mere <5% reduction in electrical conductivity. A device comprising 72 couples, fabricated through a printing process, achieved a notable voltage of 360.5 mV and a high power density of 1.278 W m^−2^ under a 50 K temperature gradient, heralding a new era of energy provisioning and sensory technology within the wearable electronics field.

## 4. Applications of Organic Thermoelectric Materials in Wearable Electronic Devices

The current mainstream applications of thermoelectric materials are energy generators, sensors, and coolers, which will be discussed below.

### 4.1. Thermoelectric Generators

Thermoelectric generators mainly have the following advantages: (1) high efficiency, as thermoelectric generators can convert low-grade heat such as waste heat or solar energy into usable electricity, and the conversion efficiency can reach 20%; (2) environmental protection, since the energy used is from low-quality heat, so it will not produce additional pollution; (3) no noise, since thermoelectric generators do not need to convert energy through mechanical movement, so no noise will be generated; and (4) reliability, as the composition of thermoelectric generators is relatively simple, and thus the failure rate is low, and long-term stable operation can be achieved.

**Figure 16:**
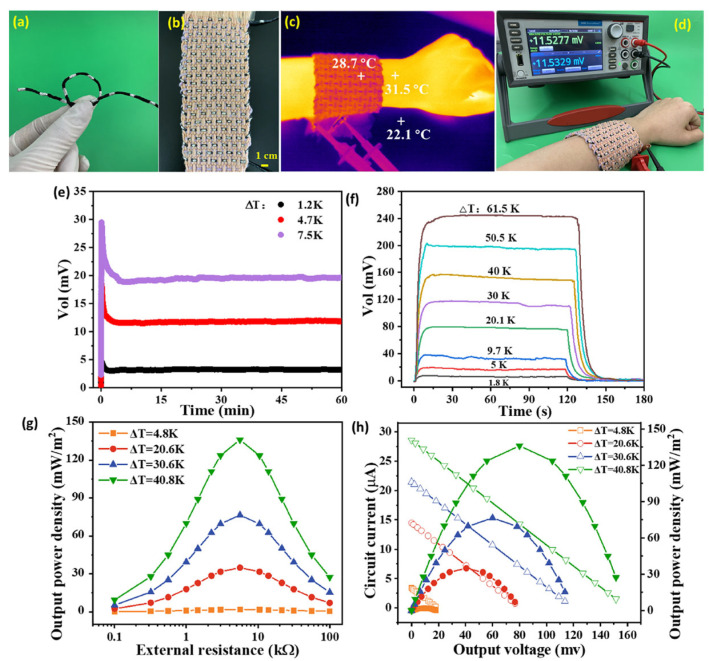
(**a**) Flexible long P/D/ED-coated yarn. (**b**) P/D/ED-coated yarn woven wearable thermoelectric wristband. (**c**) Temperature exhibition after wearing the thermoelectric device. (**d**) Thermal-voltage generated when wearing the thermoelectric device. (**e**) Thermal-voltage generated by the device within 60 min under different ΔT. (**f**) Thermal-voltage generated by the device at different ΔT values. (**g**) The relationship between the output power density of the device and external resistance at different ΔT values. (**h**) The relationship between circuit current and output power density and output voltage at different ΔT values [114].

PEDOT:PSS coatings suffer from a high modulus, low strain, and low thermoelectric properties, which hinders their application in wearable devices. Li et al. modified the PEDOT:PSS coatings on cotton yarns with DMSO and an ionic liquid to improve the flexibility and thermoelectric properties, as shown in Figure 16 [114]. In this work, a flexible continuous layer of PEDOT:PSS/DMSO/1-ethyl-3-methylimidazolium dicyanamide (P/D/ED) with a linear conformation on the surface of a yarn could significantly reduce the modules and enhance bending stability. In addition, the output power density of the composite fabric prepared from the P/D/ED-coated yarns was 136 mW m^−2^ at ΔT = 40.8 K, which was a high regain compared with previously reported wearable thermoelectric fabric devices.

**Figure 17:**
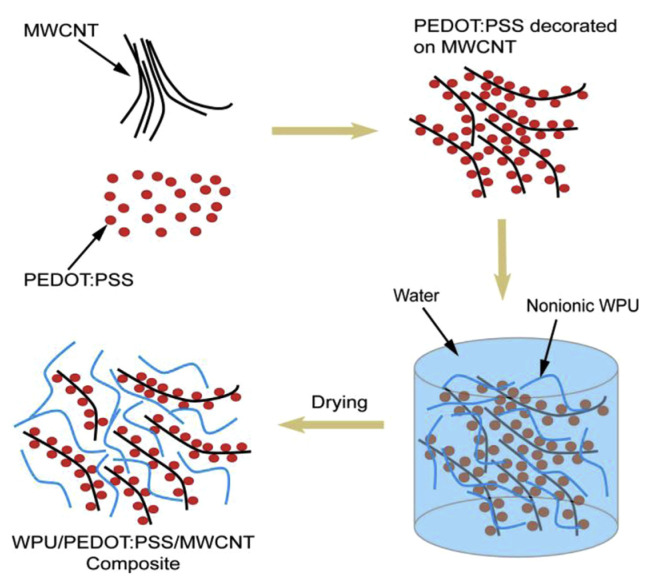
Schematic diagram of formation process of non-ionic WPU/PEDOT:PSS/MWCNT composite films [115].

To realize the wearable thermoelectric fabric, Wu et al. reported a novel water-processable thermoelectric yarn with waterborne polyurethane (WPU) as frame material, MWCNT and PEDOT:PSS as conductive and thermoelectric composite, as shown in Figure 17 [115]. The measured electrical conductivity and Seebeck coefficient was 138 S cm^−1^ and 10 μV K^−1^, respectively, leading a remarkable power factor of 1.41 μW m^−1^ K^−2^. The composite could be easily coated on commercial cotton or polyester yarns, which expanded the application scope in our daily life.

**Figure 18:**
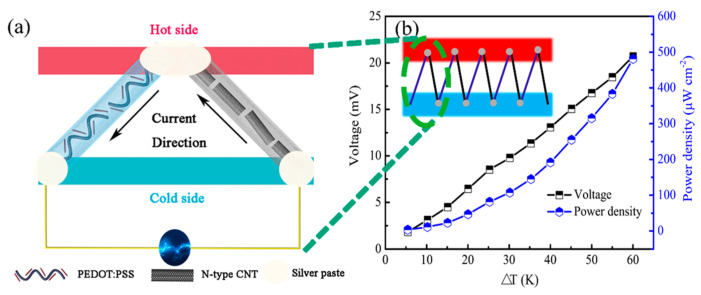
(**a**) Schematic diagram for thermoelectric device and (**b**) the output voltage and power density of five couples of legs at various temperature differences ranging from 5 to 60 K. The inset in panel b is a scheme for the fiber device consisting of five pairs of p–n legs consisting of EG–treated p–type PEDOT:PSS fibers and n–type CNT fibers [116].

Liu et al. prepared a p-type PEDOT:PSS fiber through a gelation process, and the electrical conductivity was tripled after treatment with organic solvents such as ethylene glycol and DMSO [116]. They assembled a thermoelectric device with five pairs of n-type CNT fibers and p-type PEDOT:PSS fibers, as shown in Figure 18, and the power density reached 481.17 μW m^−1^ K^−2^ at a ΔT of 60 K. This work provides a new strategy for the preparation of organic thermoelectric fibers in applications of wearable device energy harvesting.

**Figure 19:**
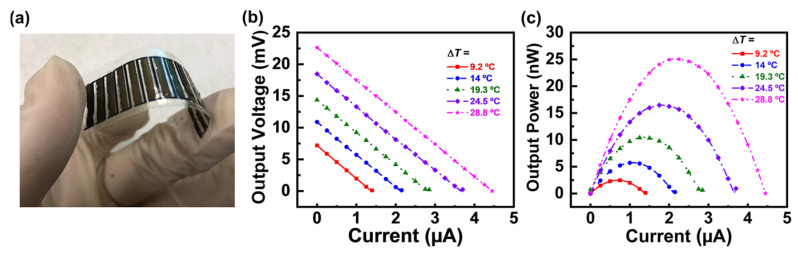
(**a**) Flexible filament-based SWCNT/P3HT thermoelectric device prepared using a full solution process. (**b**,**c**) Output voltage and output power as a function of current relative to the temperature gradient [117].

Hong et al. unveiled a novel paradigm for the construction of thermoelectric nanocomposites, leveraging solution-based methodologies coupled with biological substrates and meticulously crafted electrode patterns, aimed at enhancing the recuperation of low-tier thermal waste for wearable technologies (Figure 19) [117]. They thoroughly dispersed SWCNTs in a P3HT solution and then introduced the dispersion into silk fibroin substrates via a series of methodical spraying and vacuum-assisted procedures, culminating in an enhanced power factor higher than 200 μW m^−1^ K^−2^, an electrical conductivity near 1200 S cm^−1^, and a Seebeck coefficient around 40 μV K^−1^ at 50 °C. With the sprayed Ag NWs/PEDOT/PSS patterns as electrodes, the 14-leg in-plane SWCNT/P3HT thermoelectric generator output an open-circuit voltage higher than 22 mV and a maximum power output of about 25 nW, under a ΔT of 28.8 K. When this prototype, adorned with a silk fibroin substrate, was applied to the forearm to harness corporeal heat, it produced a V_oc_ of approximately 6.1 mV, spurred by a modest temperature differential of 8.3 K between the epidermis and the ambient atmosphere. 

**Figure 20:**
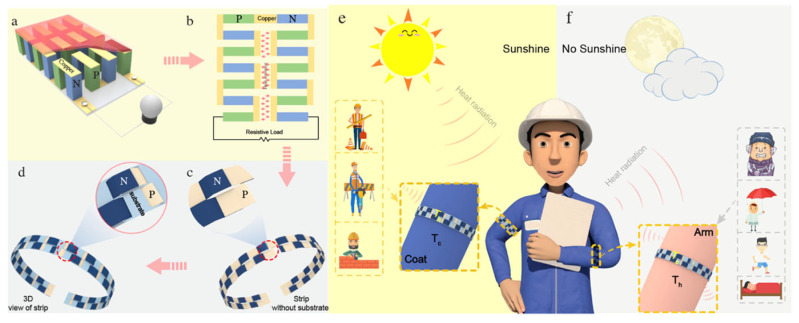
(**a**) The traditional planar structure of a thermoelectric generator; (**b**) a variant design; (**c**,**d**) schematic diagram of the photothermal fringe; (**e**,**f**) schematic diagram of the multi-purpose collector [118].

Zhang et al. fabricated dual-shell photothermoelectric textiles integrating a photo-thermal layer composed of polypyrrole (PPy) with a thermoelectric layer formed from PEDOT:Tos by employing a two-step in situ method, as shown in Figure 20 [118]. The refined photothermoelectric fabric exhibited a remarkable increase in output voltage, soaring from 294 to 536 μV under infrared illumination, with the power density reaching 13.76 nW m^−2^. Furthermore, a pliant photothermoelectric strip, comprised of the aforementioned fabric adorned with silver particulates atop a textile substrate characterized by low thermal conductivity, demonstrated varying output voltages of 2.25, 0.677, and 0.183 mV, with corresponding power outputs of 0.7031, 0.0636, and 0.0049 nW under the conditions of IR radiation, direct solar exposure, and mounted on the human arm, respectively.

**Figure 21:**
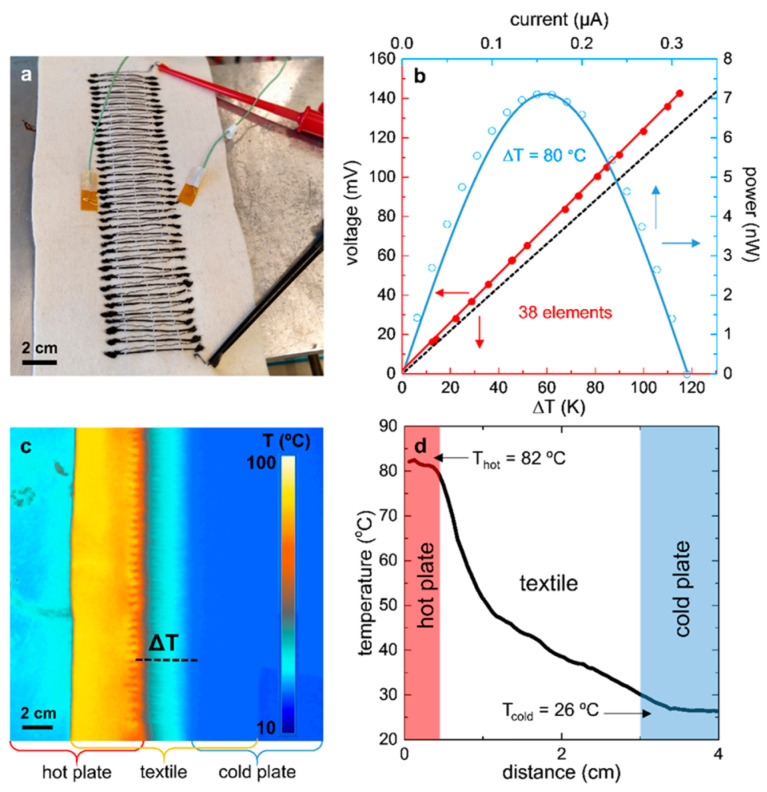
(**a**) An all-organic textile thermoelectric device with 38 n/p legs of embroidered structure; (**b**) electrical performance of the device and the relationship between the measured V_out_ and ΔT (red line); (**c**) thermal infrared image of the device in an environment of T_hot_ ≈ 82 °C and T_cold_ ≈ 26 °C; (**d**) temperature gradients of textile installations (see dashed lines in (**c**)) [119].

It is known that it is a considerable challenge to obtain air-stable n-doped organic materials because active electrons are the majority charge carriers in n-doped materials. Ryan et al. realized n-type thermoelectric yarns by coating commercial sewing threads with a composite of MWNTs and polyethylpyrrolidone (PVP), as shown in Figure 21 [119]. The electrical conductivity and Seebeck coefficient could be maintained at 1 S cm^−1^ and −14 µV K^−1^, respectively, under long-term ambient conditions (months). The prototype with 38 pairs of p–n modules (p type: PEDOT:PSS dyed silk yarn) produced a V_oc_ of 143 mV at a ΔT of 116 °C, and a maximum power output of 7.1 nW at a ΔT of 80 °C. 

Darabi et al. reported a regenerated cellulose yarn with a conductive n-type polymer, synthesized from a blend of BBL and poly(ethyleneimine) (PEI) via a meticulous spray-coating technique [120]. This strategy provides the n-type yarn with an electrical conductivity of 8 × 10^−3^ S cm^−1^ and Seebeck coefficient of −79 µV K^−1^. To enhance its durability, the yarn was enveloped with a thermoplastic elastomer coating, ensuring its resilience in atmospheric conditions for a minimum of thirteen days. Integrating the aforementioned n-type yarn with PEDOT:PSS-coated regenerated cellulose yarns resulted in commendable stability in air for no less than four days, boasting an open-circuit voltage of 1 mV for a thermal gradient of 1 K.

**Figure 22:**
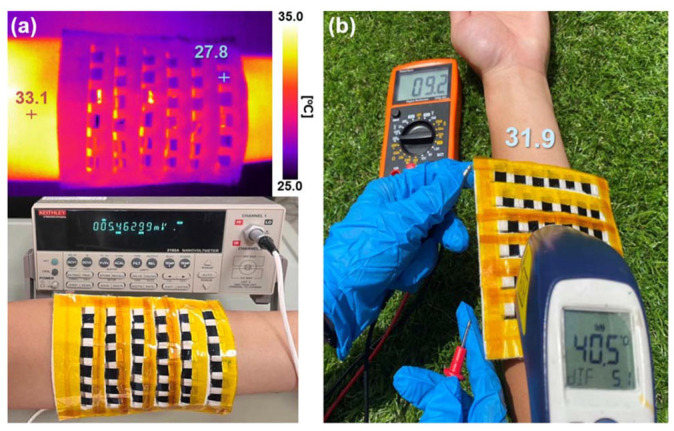
(**a**) Indoor infrared image and output thermal-voltage measurement of a PTEG worn on a human forearm; (**b**) the PTEG was draped on a human forearm and exposed to outdoor sunlight, and the PTEG output thermal-voltage and forearm temperature (31.9 °C) were recorded [121].

In the realm of photothermoelectric (PTE) materials, Tsai et al. have made significant strides. They ingeniously infused black phosphorus nanosheets, adorned with silver nanoparticles (Ag@BPs), into a PEDOT:PSS solution, bestowing the composite with photoelectric and thermoelectric functionalities [121]. With a 100 mW cm^−2^ illumination, the PEDOT:PSS/Ag@BP nanocomposite film exuded an output voltage of 528.4 µV. Further, a photothermoelectric generator (PTEG) composed of an assembly of 36 PEDOT:PSS/Ag@BP legs displayed a high electrical performance. When draped on a forearm and exposed to sunlight, the PTEG manifested an output voltage of 9.2 mV, illustrating its potential in wearable devices for energy harvesting, as shown in Figure 22.

**Figure 23:**
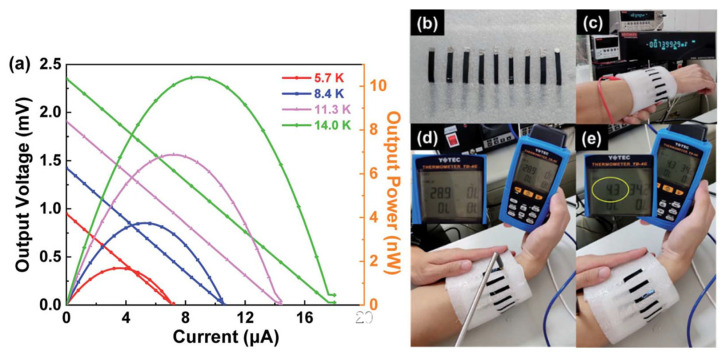
(**a**) The thermal voltage (open symbol) and power (closed symbol) of the output of a 7-leg flexible thermoelectric generator varied with the current under different temperature gradients; (**b**) photo of a 9-leg flexible thermoelectric generator module; (**c**) photos of a thermal voltage measurement with a module, which was draped on a human wrist; a thermal voltage of 0.74 mV was generated by the temperature gradient between (**d**) the environment and (**e**) the surface of the human skin [122].

Jiang et al. modulated the doping degree of PEDOT:PSS by selectively extracting the PSS component using four distinct ionic liquids (XMIM:BF_4_), each characterized by cations of varying alkyl chain lengths (with methyl, ethyl, butyl, and hexyl corresponding to X = M, E, B, and H, respectively) while maintaining an identical tetrafluoroborate BF_4_ anion [122]. It was found that incorporating smaller cations (MMIM:BF_4_) and larger ones (HMIM:BF_4_) into PEDOT:PSS films can improve the Seebeck coefficient and conductivity, respectively. The thermoelectric properties of the obtained PEDOT:PSS/MMIM:BF_4_ hybrid material was significantly improved, and the maximum power factor measured at 313 K was 86.3 μW m^−1^ K^−2^. Moreover, a robust 7-leg thermoelectric module exhibited a maximum energy output of 10.4 nW for a temperature differential of 14.0 K. A prototype of a wearable thermoelectric generator, when affixed to a human wrist, succeeded in generating a thermovoltage of approximately 0.74 mV with a temperature disparity of 4.3 K against the cooler ambient conditions, as shown in Figure 23.

**Figure 24:**
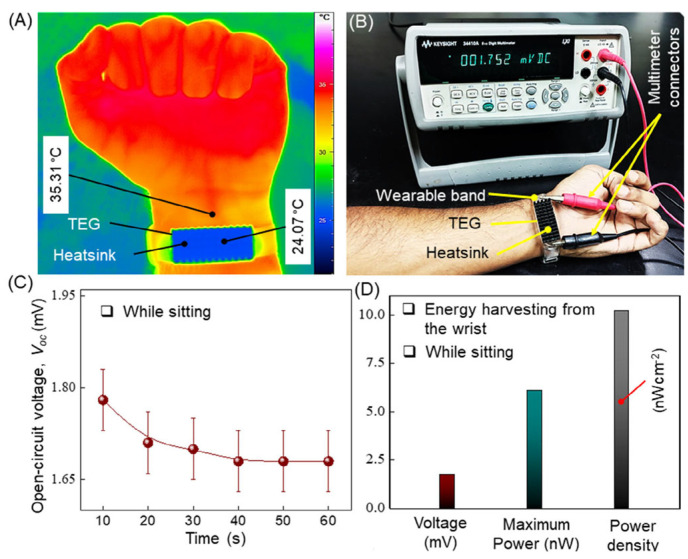
Demonstration of energy harvesting using a thermoelectric generator mounted on a wrist [123]. (**A**) Infrared thermal image of the thermoelectric generator on the wrist. (**B**) A photograph showing the developed flexible thermoelectric generator mounted on a wrist and the voltage it produces. (**C**) V_oc_ and (**D**) electrical energy generated from the wrist by the thermoelectric generator.

Hasan et al. reported a flexible and deformable thermoelectric device using a vertically aligned p-type conductive polymer (PEDOT:PSS) and n-type SWCNT films [123]. They investigated the impact of acidic post treatments and polyethylenimine (PEI) concentrations on the thermoelectric properties. The findings indicated that the power factor of DMSO/PEDOT:PSS films treated with HNO_3_ and H_2_SO_4_ reached 0.045 μW m^−1^ K^−2^ and 0.068 μW m^−1^ K^−2^, respectively. Meanwhile, PEI was used to convert SWCNT thin films from the p-type to n-type, yielding a maximum power factor of 0.15 μW m^−1^ K^−2^ when the PEI concentration was 6 wt%. The thermoelectric generator, comprising five pairs, was capable of producing an open-circuit voltage of 10.21 mV at ΔT = 80 K. When attached to a human wrist (ΔT = 11.24 K), the open-circuit voltage of the thermoelectric generator was 1.75 mV, and the maximum output power was 6.1 nW, as shown in Figure 24.

**Figure 25:**
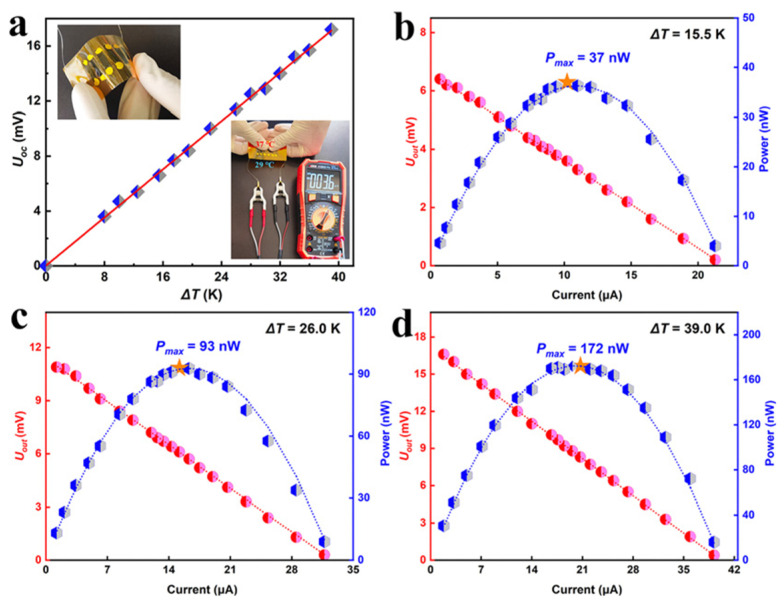
Performance of the 6-leg F-TEG fabricated with the PPy/Bi–Te/PPy nanocomposite film. (**a**) The open circuit potential (UOC) as a functionof the applied temperature difference of the as-fabricated F-TEG. (**b**–**d**)The output voltage and power versus currents at different DT = (**b**) 15.5 K, (**c**) 26.0 K and (**d**) 39.0 K [124].

Li et al. fabricated a series of sandwich-structured thermoelectric composites using an electrochemical assembly technique, as shown in Figure 25 [124]. They sequentially deposited PPy, a Bi-Te alloy, and PPy onto a stainless steel (SS) electrode. Subsequently, a PPy/Bi-Te/PPy stratified flexible nanocomposite film was procured after detachment from the SS substrate. By modulating the current density of the Bi-Te electro-deposition process and the duration of PPy electro-deposition, the composite film’s thermoelectric performance was optimized, culminating in a power factor of 244 ± 6 μW m^−1^ K^−2^, surpassing that of most composite thermoelectric materials derived via electrodeposition.

**Figure 26:**
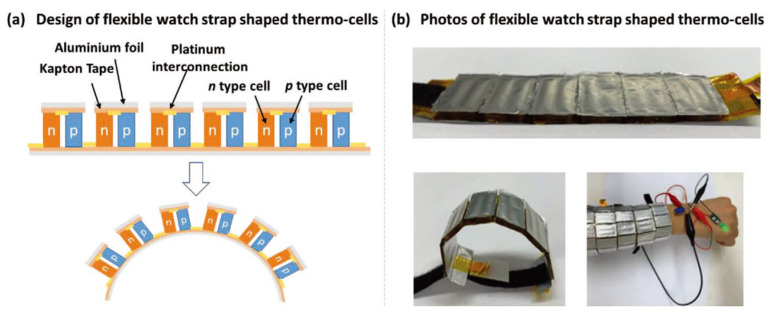
A wearable p–n thermoelectric battery. (**a**) Schematic diagram and (**b**) photo of a flexible watchband-shaped thermal battery for human heat collection [125].

In the realm of thermoelectric research, thermoelectrochemical cells have garnered attention due to their potential to deliver uninterrupted power for wearable electronics, complementing the cutting-edge flexible power storage and conversion systems. However, there still remains a dearth of in-depth studies on the optimization of wearable thermocells, particularly pertaining to the design of devices, n-type electrolyte selection, and the amalgamation of electrodes with electrolytes. Liu et al. crafted an elastic and stretchable n-type gel electrolyte comprising polyvinyl alcohol-FeCl_2_/_3_ through an in situ chemical cross-linking process and incorporated this electrolyte into a 3D porous PEDOT:PSS electrode to form an n-type thermoelectrochemical cell, as shown in Figure 26 [125]. This n-type cell showed an exceptional Seebeck coefficient of 0.85 mV K^−1^ and remarkable output current density of 1.74 A m^−2^ K^−1^. When combined with an optimized p-type cell consisting of a carboxymethyl cellulose–K_3/4_Fe(CN)_6_ electrolyte and 3D PEDOT:PSS edge-functionalized graphene/carbon nanotube electrodes (−1.22 mV K^−1^ and 1.85 A m^−2^ K^−1^), the device, featuring 18 pairs of p–n cells, generated an output voltage of 0.34 V at ΔT = 10 K. This serially connected device was use to make a prototype watch strap that was capable of harnessing body heat to charge a supercapacitor (up to 470 mF) and power a green light-emitting diode, thus showcasing its practical utility.

**Figure 27:**
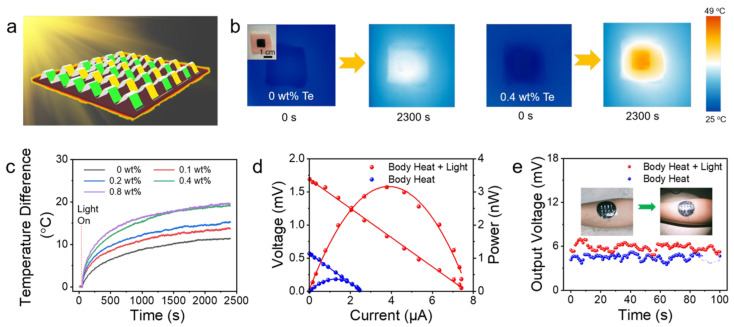
Output properties of the WTEGs when exposed to light. (**a**) Schematic illustration of enhancing the output property through the photothermal effect. (**b**) Top-view infrared thermal images of photothermal conversion by Te/TPU films with a 0.4 wt % Te composition compared to unadulterated TPU films under an illumination intensity of 1000 W m^−2^, with an inset portraying the Te/TPU film affixed to porcine dermis. (**c**) Dynamic changes in temperature differential for varying masses of Te NWs under the same light intensity of 1000 W m^−2^, with the baseline temperature set at ambient conditions (25 °C). (**d**) The characteristic curves of voltage/power versus current in scenarios with and without the specified light intensity. (**e**) Output voltage of eight pairs of thermoelectric units adjoined to a human forearm, pre- and post-irradiation, in an ambient temperature of 19.5 °C [126].

Chen et al. engineered a nanowire (NW) thin film with a sinuous architecture that harnesses the photothermal properties of Te NWs to provide a source of heat, while employing heterojunctions of p–n NWs (n-type Ag_2_Te and p-type Cu_1_._75_Te NWs) as the thermoelectric constituents, as shown in Figure 27 [126]. Under light irradiation, the device achieved not only an output voltage, but also personal thermal management, thereby ensuring that the epidermis remains tepid and comfortable amid frigid climates. Moreover, the material possessed considerable stretchability and can be easily applied to wearable devices.

**Figure 28:**
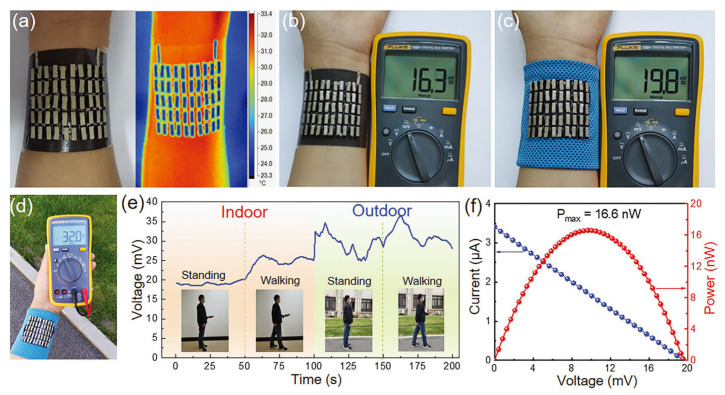
(**a**) Photo of an OTEG adhered to a human wrist alongside the corresponding thermal distribution infrared camera image at a steady temperature of 23 °C. (**b**) OTEG’s Voc measured under the conditions in panel (**a**). (**c**) V_oc_ of the OTEG integrated into a wristband at the same temperature and static conditions. (**d**) V_oc_ measured outdoors at 21 °C with wind influence. (**e**) Continuous monitoring of V_oc_ of an individual donning the OTEG, whether stationary or ambulatory, and indoors or outdoors. (**f**) The outputs of the OTEG worn on the wrist of a stationary subject [127].

Due to the brittleness of some thermoelectric materials, researchers encapsulate them in an elastic material to prepare flexible composite materials, but the overall thermal resistance will increase, resulting in a low heat transfer efficiency and low thermoelectric conversion efficiency. Guided by finite element analyses, Wang et al. found that Cu-coated polydimethylsiloxane possessed high thermal conductivity. As a result, the end of a thermoelectric material that is supposed to contact hot surfaces could receive more heat, and thus maximized the temperature difference utilization ratio to 86%, which was 75.5% higher than that of a routine organic thermoelectric generator, as shown in Figure 28 [127]. They then simulated and tested the output performance of the integrated 50 pairs of p/n porous polyurethane/SWCNT thermoelectric legs onto the designed substrate under different thermal conditions, and the results were consistent with the simulations. When the device was applied to human skin at room temperature (23 °C) and a ΔT of 6 °C without wind, the output voltage reached 16.3 mV. In windy conditions, the output power reached 32 mV with a maximum output power of 16.6 nW.

**Figure 29:**
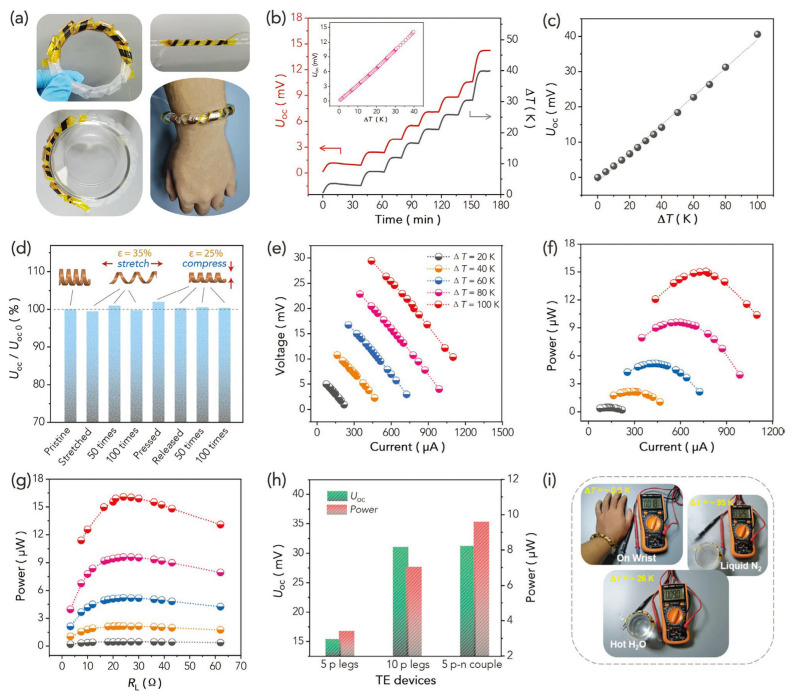
(**a**) Photos of the reported flexible TEG device. (**b**) Generated thermal-voltage as a function of time at different ΔT values. (**c**) Uoc generated by the flexible TEG at different ΔT values. (**d**) The U_oc_ variation of the TEG under influence of external forces at a ΔT of 50 K. (**e**,**f**) The generated voltage and power as a function of the current at ΔT values ranging from 20 to 100 K. (**g**) The output power as a function of external load resistance at ΔT values ranging from 20 to 100 K. (**h**) Comparison of the output performance of the three fabricated TEGs at a ΔT of 80 K. (**i**) Photographs showing the potential applications for the TEG for heat energy harvesting [128]. The different colors in (**f**,**g**) correspond to the explanation in (**e**).

Liang et al. synthesized a flexible PEDOT-Tos/Te/SWCNTs ternary nanocomposite film, characterized by a distinctive stratified structure, which demonstrated a significantly enhanced power factor of 131.9 ± 8.5 μW m^−1^ K^−2^, 120 times higher than a pure PEDOT-Tos film [128]. A pliable, extensible, and compressible helical 3D thermoelectric generator comprised of these nanocomposite films was devised to proficiently transmute thermal energy into electrical power via a transverse thermal gradient, as shown in Figure 29. In prototypes of the TEG equipped with 10 p-type limbs and five pairs of p–n junctions at a ΔT of 80 K, the generated output power reached an impressive 7.04 and 9.59 µW, respectively. Moreover, this helical device can be employed to capture human body heat from the wrist, producing electricity from the temperature variances induced by thermal entities such as hot water and liquid nitrogen.

**Figure 30:**
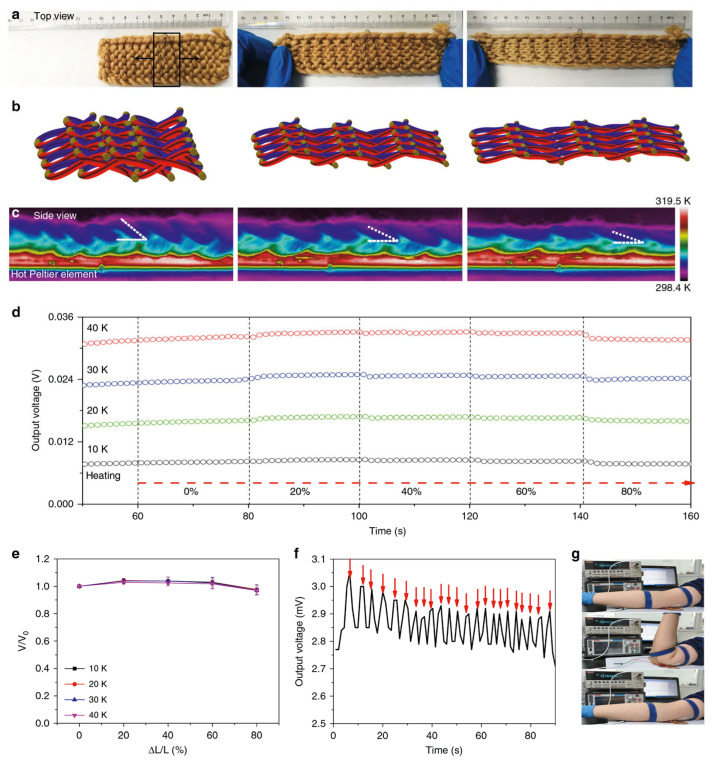
A reported TEG device with good stretchability. Photographs of (**a**) the top view and (**b**) schematic after longitudinal stretching by 0%, 40%, and 80%. (**c**) Infrared thermal images of the side view when contacting a Peltier element (~318.1 K). (**d**) Output voltage responses of the TE device stretched to 80% at different ΔT values. (**e**) TE performance degradation versus longitudinal strain. (**f**) Plot of the real-time output voltage responses of TE devices attached to a moving elbow. (**g**) Photos showing the elbow movement corresponding to plot (**f**) [129].

Sun et al. prepared stretchable 3D free-standing thermoelectric generators by utilizing interlocked thermoelectric modules and interlaced thermoelectric modules composed of carbon nanotube fibers doped in an alternating pattern and wrapped with acrylic fibers, as shown in Figure 30 [129]. This fabric generator, boasting a maximum power density of 70 mW m^−2^ with a temperature gradient of 44 K, exhibited exceptional stretchability (approximately 80% strain) without compromising its power output. This innovation marks the inaugural incorporation of thermoelectric modules into truly pliable textiles that conform to bodily motions, eschewing the need to embed them within garments without any sacrifice in flexibility or power, which holds profound implications for wearable technologies.

### 4.2. Thermoelectric Sensors

The advantages of thermoelectric sensors include a wide measuring range; high temperature precision up to 0.1 °C; fast response speed of up to a few milliseconds in response to temperature changes; strong reliability; long durability; strong anti-interference ability with ignorable effect from external magnetic field; and electromagnetic interference.

**Figure 31:**
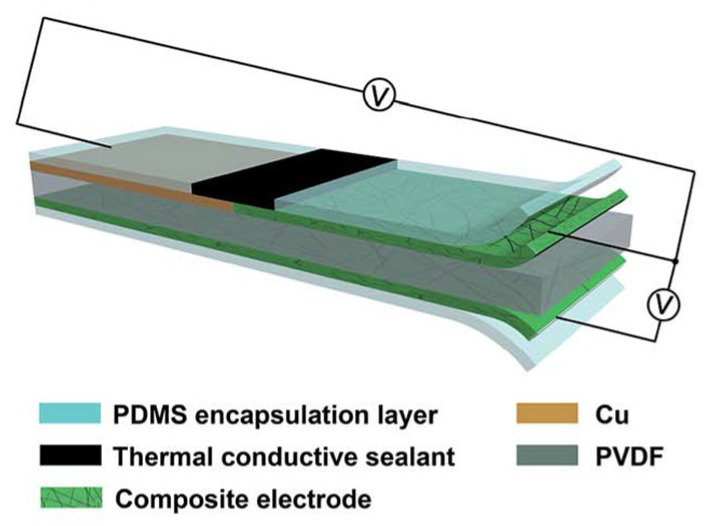
Structure diagram and testing principle of temperature-sensitive piezoelectric device [130].

Zhu et al. developed a flexible, sandwich-structured, dual temperature and physiological parameter sensor based on piezoelectric poly(vinylidene fluoride)(PVDF) and thermoelectric PANI, as shown in Figure 31 [130]. In this device, conducting PANI polymer film electrodes contribute a higher electromechanical conversion than traditional silver electrodes. This device is not only sensitive to physiological signals such as human motion, but also sensitive to ambient temperature with a high sensitivity of 45.5 μV K^−1^ and a quick response time of 1.2 s.

**Figure 32:**
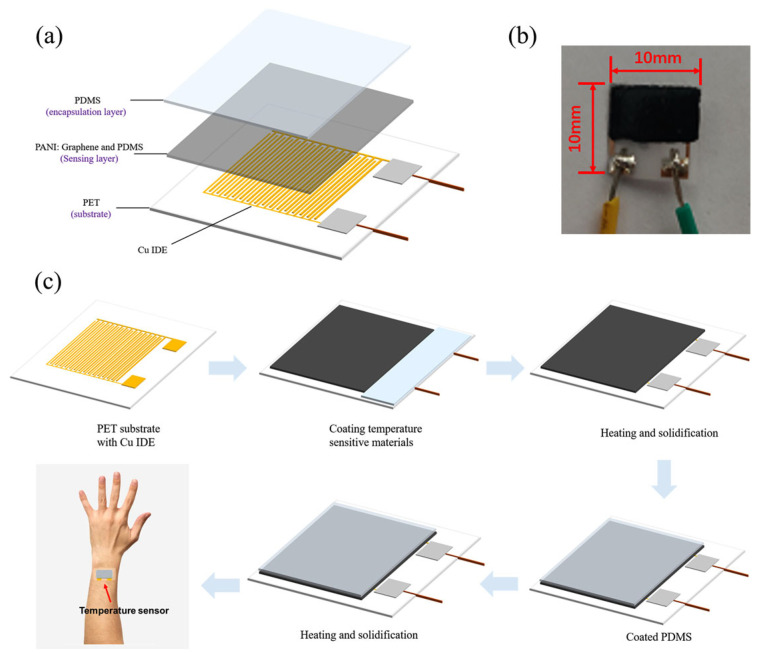
An ultrahigh linear sensitive temperature sensor based on PANI preparation. (**a**) Schematic diagram of the temperature sensor structure. (**b**) Optical image of the temperature sensor. (**c**) Fabrication process of the skin-attachable temperature sensor [131].

Liu et al. reported a hybrid temperature sensor based on PANI containing graphene and PDMS, as shown in Figure 32 [131]. The sensor exhibited a high sensitivity of up to 1.60% °C^−1^, extraordinary linearity with an R^2^ = 0.99, a high accuracy of 0.3 °C, and a quick response time of 0.7 s in a temperature range of 25–40 °C. The hybrid sensor can monitor real-time temperatures and respiratory rates when combined with a read-out circuit and filter circuit with an analog/digital converter, presenting great potential for its use in medical diagnosis and monitoring.

**Figure 33:**
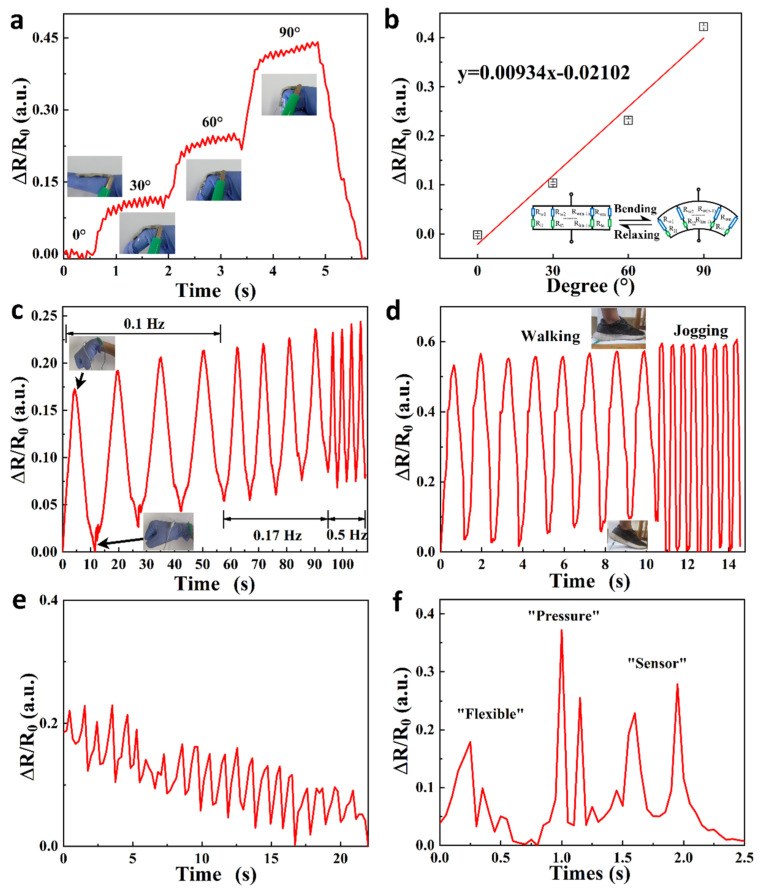
Testing of a flexible, wearable, piezoresistive sensor fabricated using a PPy/reduced graphite oxide aerogel film (**a**–**e**) under large deformation: (**a**) finger bending, (**b**) wrist bending, (**c**) equivalent circuit, and (**d**) shoe sole test; and under small deformations: (**e**) pulse sensing and (**f**) voice sensing [132].

Wei et al. developed a facile sol–gel-hydrothermal reduction method and prepared a PPy/reduced graphite oxide aerogel (PPy@rGA) composite film, which was very suitable for making a flexible wearable piezoresistive sensor, as shown in Figure 33 [132]. The presence of PPy nanoparticles among the rGO sheets created more conductive paths under external pressure, affecting the resistance and resulting in better piezoresistive sensing performance. Specifically, the hybrid film-based piezoresistive sensor with an optimized mass ratio of pyrrole monomer/graphene oxide of 2:1 exhibited a sensitivity of 0.9 kPa^−1^ under a pressure from 0 to 1 kPa, with a short response time of 165 ms, relaxation time of 132 ms, and long-term stability up to 10,000 cycles.

**Figure 34:**
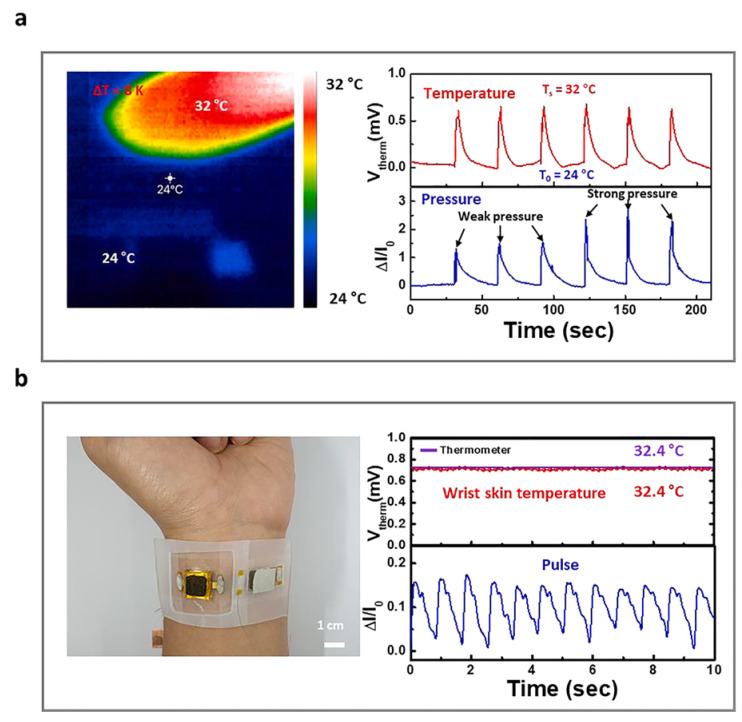
(**a**) Simultaneous detection of applied pressure and temperature using a dual-mode sensor. (**b**) Wrist pulse and skin temperature detected using an integrated band of pressure and temperature sensors [133].

Jang et al. also fabricated a pressure and temperature sensor using elastic carbon foam coated with the conducting polymer P3HT, as shown in Figure 34 [133]. This sensor exhibited a sensitivity to pressures up to 102.4 kPa^−1^ (<1 kPa), a fast response time of 50 ms, and a high lifetime of over 10,000 cycles of pressure loading and unloading. Owing to the P3HT, this sensor also showed good thermoelectric properties with a Seebeck coefficient of 82.5 μV K^−1^ in the near-body-temperature range (25–55 °C), making it an excellent biosensors for bio-signals such as arterial pulses, swallowing, and speech as these signals could be distinctively detected when the sensor was attached to the skin.

**Figure 35:**
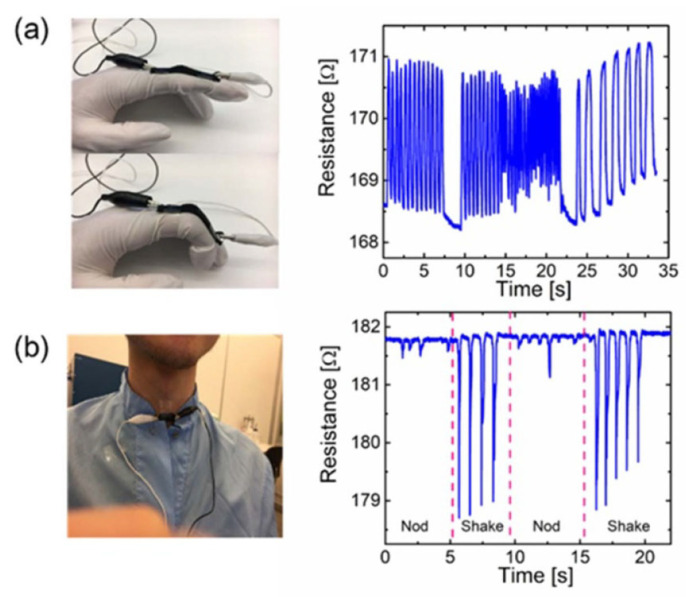
Silk polypyrrole hydrogels for human motion detection. Real-time (**a**) finger flexing–stretching periodicity and (**b**) head nodding/shaking electrical signals [134].

Han et al. prepared a conductive hydrogel through in situ polymerization of pyrrole in the presence of a silk fibroin network, as shown in Figure 35 [134]. The obtained hydrogel presented a high electrical conductivity (26 S m^−1^) and fast response to conformational changes. The flexible, wearable strain sensors made using the hydrogel could detect both large and subtle human motions and were thus proposed for the monitoring of various body movements.

**Figure 36:**
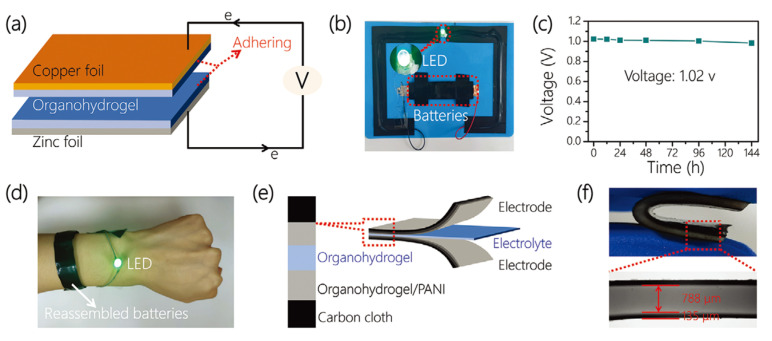
Structures, performance, and applications of removable batteries and all-in-one supercapacitors. (**a**) Schematic diagram of a removable battery structure. (**b**) A demonstration of the hydrogel batteries powering an LED. (**c**) Battery voltage as a function of time. (**d**) Application of the batteries in wearable electronics. (**e**) Schematic diagram of integrated supercapacitor structure. (**f**) Details of the sandwich-like organic supercapacitor [135].

Inspired by the molecular structure of glutinous rice, Zhou et al. designed an organic hydrogel to introduce amylopectin into the copolymer network in a glycerin/water solvent containing sodium chloride as a conductive component through a “one-pot” crosslinking process, so as to achieve a hydrogel with excellent conductivity, tensile properties, tensile strength, adhesion, frost resistance, and moisture retention, as shown in Figure 36 [135]. The wearable device assembled from the hydrogel had the characteristics of a wide operating range, high sensitivity (measuring factor: 8.82), and short response time. It is worth noting that the device could maintain a relatively good response in a relatively harsh environment.

**Figure 37:**
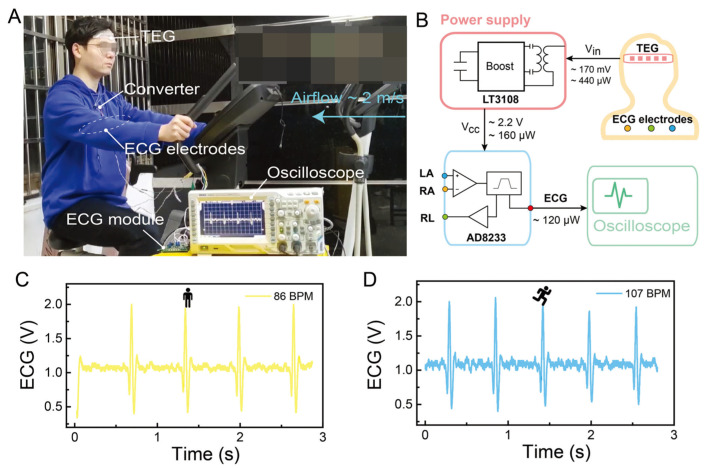
Demonstration of self-powered Electrocardiograph (ECG) system using s-TEG. (**A**) Photograph of the experimental setup used to drive the ECG module utilizing human body heat. (**B**) Functional block diagram of the self-powered ECG operating system. ECG signal at (**C**) rest and (**D**) after cycling [136].

Fan et al. obtained a 3D p–n array by repeatedly cutting and gluing Bi_2_Te_3_ materials, subsequently depositing Au on it by magnetron sputtering, connecting p–n units in series, and finally depositing Ni on the material to reduce the resistance of the material (the material contains 8 pairs of p–n legs), as shown in Figure 37 [136]. The obtained device had a power density of 13.8 μW cm^−2^ at room temperature (15 °C), and the output power increased significantly to 71.8 μW cm^−2^ at an air speed of 2 m s^−1^. The experimental results showed that the device can stably supply power to an electrocardiogram (ECG) module in real time without an additional power supply.

## 5. Conclusions and Prospects

The environmentally friendly thermoelectric conversion mechanism and unique advantages of organic thermoelectric materials are widely used in WEDs, among which, electronic-type organic thermoelectric materials have been more researched; these include p-type organic thermoelectric materials with excellent thermoelectric properties including PEDOT, P3HT, etc. Excellent n-type electronic-type organic thermoelectric materials include PBFDO, BLL, etc. As an emerging material, ionic organic thermoelectric materials have a broad research prospect and are likely to open up new development directions in the field of organic thermoelectrics. However, currently, there are more p-type ionic organic thermoelectric materials, while n-type materials are more scarce, which is due to the fact that cations are usually more mobile than anions in electrolytes. In the future, if we want to develop higher output ionic organic thermoelectric devices, we will inevitably have to construct p–n structures, so the development of new n-type ionic organic thermoelectric materials is an urgent task.

The current research on organic thermoelectric materials is mainly focused on conducting polymers, but we should also explore the potential of organic small molecules, complexes, and other materials in the field of thermoelectrics, not only to develop new organic thermoelectric materials with higher thermoelectric properties, but also to explore the mechanism and optimize the performance of the original materials, so that organic thermoelectric material systems are more abundant and more mature. Although the thermoelectric transport mechanism of nanoscale thermoelectric devices is still in the preliminary stage of exploration, the moment that the mass production of nanoscale thermoelectric devices becomes a reality, it can bring the performance of thermoelectric devices inside WEDs to another level.

## Figures and Tables

**Table 1:** Comparison of properties of some common organic thermoelectric materials.

Material	S	σ	PF	Ref.
	µV/K	S/cm	µW/m K^2^	
PBFDO	−21	2000	90	[80]
PEDOT:Tos	117	923	1270	[82]
PEDOT:PSS	72.6	890	469	[83]
PEDOT:PSS (H_2_SO_4_ and NaOH post treatments)	39.2	2170	334	[84]
PEDOT:PSS (a solution of 0.1 M MAl in 80% DMF/20% water)	28	1831	144	[85]
PEDOT:PSS (IL treatment)	17	520	15	[86]
PEDOT:PSS (IL and acid-treated, then base-treated)	1594.8	63.3	754	[87]
PEDOT:OTf (NaOH treatment)	2342 ± 98	49.2	568 ± 64	[88]
PEDOT:NWs (H_2_SO_4_ and NaOH treatments)	715.3	25.5	46.51	[89]
SWCNT/PANI (ethanol treatment)	2356	39.2	362	[90]
P3HT	39.5	2	31	[91]
PPy nanowires (chemical oxidation polymerization)	2.22 ± 0.3	10.1 ± 0.1	2.26 ± 0.36	[92]
PPy nanotube (chemical oxidation polymerization)	33.82	12.76	0.55	[93]
PPy/rGO (template-directed in situ polymerization)	41.6	26.9	3.01	[94]
PPy/MWCNTs (68 wt%) (in situ polymerization)	35–40	24.4	2.2	[95]
PPy/SWCNTs (interfacial polymerization)	47 ± 34.2	33.2 ± 0.7	37.6 ± 2.3	[96]
PDPF	−235	1.35	4.65	[97]
PDPH	−87	1.01 × 10^−4^	5.11 × 10^−4^	[97]
BBL	−101	0.42	0.43	[97]
FBDPPV	−210	14	25.5	[97]
BDPPV	−320	0.26	1.6	[97]

## Data Availability

Data availability is not applicable to this article as no new data were created or analyzed in this study.
